# Optoelectronic performance prediction of HgCdTe homojunction photodetector in long wave infrared spectral region using traditional simulations and machine learning models

**DOI:** 10.1038/s41598-024-79727-y

**Published:** 2024-11-15

**Authors:** Shonak Bansal, Arpit Jain, Sandeep Kumar, Ashok Kumar, Parvataneni Rajendra Kumar, Krishna Prakash, Mohamed S. Soliman, Mohamed Shabiul Islam, Mohammad Tariqul Islam

**Affiliations:** 1 Department of Electronics and Communication Engineering, Chandigarh University, Gharuan, Punjab India; 2 Department of Computer Science and Engineering, Koneru Lakshmaiah Educational Foundations, Vijayawada, India; 3 School of Computer Science and Artificial Intelligence, SR University, Warangal, India; 4 School of Computing, DIT University, Dehradun, Uttarakhand India; 5 Department of AI&ML, NRI Institute of Technology, Agripalli, Eluru, AP 521212 India; 6 Department of Electronics and Communication Engineering, NRI Institute of Technology, Agripalli, Eluru, AP 521212 India; 7 Department of Electrical Engineering, College of Engineering, Taif University, 21944 Taif, Saudi Arabia; 8 Department of Electrical Engineering, Faculty of Energy Engineering, Aswan University, Aswan, 81528 Egypt; 9 Faculty of Engineering (FOE), Multimedia University (MMU), 63100 Cyberjaya, Selangor Malaysia; 10 Department of Electrical, Electronic and Systems Engineering, Faculty of Engineering and Built Environment, UKM, 43600 Bangi, Selangor Malaysia

**Keywords:** HgCdTe, Homojunction, Infrared photodetector, Long-wavelength infrared (LWIR), Machine learning, Noise equivalent power, Optoelectronic, Photodetector, Regression, Silvaco TCAD, Quantum efficiency, Optics and photonics, Optical materials and structures, Other photonics, Software, Materials for optics

## Abstract

This research explores the design of an infrared (IR) photodetector using mercury cadmium telluride (Hg_1–*x*_Cd_*x*_Te). It proposes two- and three-dimensional homojunction models based on p^+^-Hg_0.7783_Cd_0.2217_Te/n^–^-Hg_0.7783_Cd_0.2217_Te, focusing on applications in the long-wavelength infrared range. The photodetector’s performance is analyzed using Silvaco ATLAS TCAD software and compared with analytical calculations based on drift-diffusion, tunneling, and Chu’s approximation techniques. Optimized for operation at 10.6 μm wavelength under liquid nitrogen temperature, the proposed photodetector demonstrates promising optoelectronic characteristics including the dark current density of 0.20 mA/cm^2^, photocurrent density of 4.98 A/cm^2^, and photocurrent density-to-dark current density ratio of 2.46 × 10^4^, a 3-dB cut-off frequency of 104 GHz, a rise time of 0.8 ps, quantum efficiency of 58.30 %, peak photocurrent responsivity of 4.98 A/W, specific detectivity of 3.96 × 10^11^ cmHz^1/2^/W, and noise equivalent power of 2.52 × 10^–16^ W/Hz^1/2^ indicating its potential for low-noise, high-frequency and fast-switching applications. The study also incorporates machine learning regression models to validate simulation results and provide a predictive framework for performance optimization, evaluating these models using various statistical metrics. This comprehensive approach demonstrates the synergy between advanced materials science and computational techniques in developing next-generation optoelectronic devices. By combining theoretical modeling, simulation, and machine learning, the research highlights the potential to accelerate progress in IR detection technology and enhance device performance and efficiency. This multidisciplinary methodology could serve as a model for future studies in optoelectronics, illustrating how advanced materials and computational methods can be utilized to enhance device capabilities.

## Introduction

The field of infrared (IR) sensing has made remarkable advancements, driven by the demand for photodetectors with enhanced sensitivity, wider spectral coverage, and enhanced operational efficiency. Among the various materials investigated for IR photodetection^1–6^, mercury cadmium telluride (Hg_1–*x*_Cd_*x*_Te) has emerged as a leading candidate for next-generation high-performance IR photodetectors. Its prominence arises from a combination of favorable properties, including an adjustable bandgap, low leakage current, high photon absorption coefficient, improved stability, reduced thermal generation rate, moderate dielectric constant for minimal device capacitance, superior lattice matching for high-quality crystal growth, low thermal expansion coefficient for device stability, and advantageous optoelectronic characteristics^7–14^.

IR photodetectors have found widespread applications across various sectors. These include military uses, optical communications, civilian applications, thermal and biomedical imaging, remote sensing, missile guidance systems, fire detection, gas sensing, satellite-based remote sensing, motion detection, spectroscopy, surveillance, chemical analysis, telecommunication systems, and night vision technology^15–19^. The versatility and effectiveness of IR photodetectors in these diverse fields underscore their significance in modern technological advancements.

The compound Hg_1–*x*_Cd_*x*_Te can be epitaxially grown by combining HgTe and CdTe. HgTe is semi-metallic with zero bandgap and low resistivity, while CdTe is a semiconductor featuring a bandgap of 1.6 eV. Their similar lattice constants allow defect-free growth of Hg_1–*x*_Cd_*x*_Te at any cadmium (Cd) composition. The minimal lattice constant variation with Cd composition enables high-quality layers and heterostructures. This combination offers bandgaps ranging from 0 to 1.6 eV. Hg_1–*x*_Cd_*x*_Te maintains a zinc blende structure across all Cd compositions, like its constituents^8^. The tunable bandgap of Hg_1–*x*_Cd_*x*_Te through Cd composition has led to various high-performance IR photodetectors, including p-n^9,18,20–24^, p-i-n^23,25^, avalanche^26^, barrier^11–13,27–29^, and dual-band configurations^10,16^, operating at both cryogenic and room temperatures. However, cryogenic cooling increases power consumption, cost, and weight. Hg_1–*x*_Cd_*x*_Te technology primarily serves military-related applications, particularly in specific conditions or object detection. Despite its advantages, Hg_1–*x*_Cd_*x*_Te has drawbacks such as weak Hg-Te bonding, causing instability in bulk, surface, and interfaces. Yield and uniformity concerns persist, especially in the long-wavelength IR (LWIR: 8–12 μm) region^30^. The LWIR spectral region shows promise for applications in free-space optical communication systems, offering several advantages over traditional communication methods^18,24,32–34^. The LWIR region contains two strategically important atmospheric windows at 9.6 and 10.6 μm, which have attracted significant research attention for developing free-space optical communication systems. The 10.6 μm window is particularly noteworthy for its resistance to adverse weather conditions such as haze and fog. This characteristic makes the 10.6 μm wavelength within the LWIR range especially valuable for applications requiring reliable performance in challenging atmospheric environments^24^. LWIR detectors based on intersubband transitions in III–V semiconductor heterostructures^34–37^ offer minimal dark current but fall short in overall performance compared to competing technologies. Hg_1–*x*_Cd_*x*_Te remains the dominant material for IR photodetectors, outperforming alternatives in sensitivity and quantum efficiency in the LWIR region when used as the absorber/active layer. However, its primary limitation is the high dark current caused by Auger recombination, necessitating low-temperature operation^38,39^. To address this, researchers are exploring strategies such as band structure engineering using Type-II superlattices^29^, which aim to suppress Auger recombination by modifying energy levels, particularly at cryogenic temperatures. Other approaches include the complex graded gap and doping multilayer devices^12^, the integration of two-dimensional (2D) materials such as graphene^19,22,23,40^ and black phosphorus^41^ with HgCdTe, as well as the nBn and pBp device architectures, which incorporate a wide-bandgap HgCdTe barrier layer between narrow-bandgap layers to reduce dark current and enable higher temperature operation^22,28,42^. Additionally, the pBp-avalanche photodiode^43^ is being explored for its potential to further enhance performance, particularly in high-sensitivity and high-temperature detection applications. These techniques focus on enhancing Hg_1–*x*_Cd_*x*_Te-based photodetectors to deliver improved performance at or near room temperature while maintaining their superior characteristics in the LWIR region.

A significant challenge in advancing Hg_1–*x*_Cd_*x*_Te technology lies in its integration with CMOS process technology, primarily due to its incompatibility with silicon-based manufacturing processes. This incompatibility presents a major obstacle to seamless integration with existing semiconductor fabrication techniques. Nevertheless, despite this limitation in silicon-based CMOS compatibility, researchers and engineers continue to show considerable interest in the Hg_1–*x*_Cd_*x*_Te/CdZnTe interface^24^. This interest stems from the excellent lattice matching between these materials, which offers potential photodetector performance and fabrication advantages. The exploration of this interface could potentially lead to novel device architectures and improved performance characteristics, making it a key area of interest for ongoing research and development efforts in the field of IR photodetector technology.

The development of high-performance photodetectors, including those based on Hg_1–*x*_Cd_*x*_Te, presents significant challenges, largely due to the complex interaction of numerous device parameters. This process necessitates extensive experimentation with various aspects such as device geometry, material composition, and doping profiles. Each of these parameters requires thorough validation and optimization, contributing to the complexity of the design process. Accurate performance estimation of photodetectors demands advanced modeling techniques due to the sophisticated relationships between the device’s structural components, material properties, and optoelectronic characteristics. The dependency on these factors creates a diverse design space, where changes in one parameter can have significant effects on others. This complexity underscores the need for robust simulation tools and comprehensive analytical approaches to navigate the design process effectively and predict device performance with high accuracy.

Although Hg_1–*x*_Cd_*x*_Te-based photodetectors have seen substantial performance enhancements, particularly in the LWIR spectrum, the incorporation of machine learning approaches offers the potential to expedite their development further. These computational techniques can offer predictive capabilities and minimize dependence on expensive and time-consuming experimental procedures, thereby streamlining the overall design process.

Traditional mathematical approaches often may prove inadequate when dealing with extensive and complex datasets typical in advanced photodetector research. This limitation has led to the increasing use of machine learning (ML) methods, a branch of artificial intelligence, which are well-known for their adaptability in evaluating and predicting material characteristics and device performance across a wide range of conditions and configurations^44,45^. Reproducing the experimental design of complex systems typically demands the development of sophisticated frameworks, which can be time-consuming and resource-intensive. ML techniques offer a solution to these challenges by efficiently identifying key parameters and determining unknown parameter values, substantially decreasing the time and effort needed for analysis and prediction in photodetector research. ML algorithms can process large datasets to develop and refine predictive models, thereby improving decision-making and forecasting accuracy in device performance. Regression analysis, a key ML technique, is particularly useful in quantifying relationships between variables in photodetector design, such as how changes in applied voltage or incident light wavelength influence the device’s electrical and optical responses. ML regression models have rapidly emerged as essential tools in nanomaterials and materials science^46,47^ demonstrating remarkable proficiency in making accurate predictions from complex device and material data. A ML algorithm was proposed to improve quadrant photodetectors’ measurement range and accuracy^48^. By applying the Ridge regression technique, the algorithm significantly enhanced measurement precision, showing a substantial improvement in accuracy compared to traditional methods. Likewise, the ML model has been utilized to predict the responsivity and detectivity of 2D metal halide perovskite photodetector. The model was confirmed through actual experimental data, demonstrating minimal residual error and offering valuable insights into the application of ML for a more rational understanding of photodetector performance^49^. These advancements reflect the broader trend in utilizing machine learning for optimizing device performance. Recent studies highlight this trend across various contexts, including near-IR organic semiconductors^50^, the structural optimization of silicon-based devices using algorithms like light gradient boosting machine (LGBM) and extreme gradient boosting (XGBoost)^51^, and methodologies that employ artificial neural networks (ANN) to predict the responsivity of challenging materials such as borophene^52^. Additionally, ML regression models’ ability to rapidly and accurately predict bandgaps has become crucial for designing high-performance photodetectors, enabling efficient screening of polymers and other materials before experimental synthesis^53^. These ML models can uncover deeper insights into optoelectronic device behavior and underlying patterns that may not be immediately apparent through conventional methods, effectively revealing the relationships governing photodetector properties and performance metrics. Consequently, ML proves to be an ideal tool for exploring and refining photodetector designs, simplifying the process of predicting device properties without the need for computationally demanding theoretical calculations.

This paper presents a comprehensive 2D and three-dimensional (3D) architecture model of a p^+^-Hg_0.7783_Cd_0.2217_Te/n^–^-Hg_0.7783_Cd_0.2217_Te homojunction photodetector for the LWIR spectral region. The proposed LWIR photodetector is designed to operate at a wavelength of 10.6 μm under cryogenic or liquid nitrogen temperature (77 K). The numerical simulation of the photodetector is analyzed using Silvaco ATLAS TCAD software, with results compared to analytical models based on drift-diffusion, tunneling, and Chu’s approximation techniques. The simulation incorporates all relevant recombination processes and examines various electrical and optical parameters. Electrical characterization includes energy bandgap, electric field profile, current densities, resistance-area product, frequency response, and photoswitching response. Optical characterization focuses on quantum efficiency, photocurrent responsivity, specific detectivity, and noise equivalent power. This comprehensive approach aims to thoroughly understand the photodetector’s behavior and performance characteristics, validating the simulation results against analytical expressions and exploring the photodetector’s potential for optical communication applications. To demonstrate the performance limitations of the HgCdTe-based LWIR photodetector, current density under dark and illumination conditions is evaluated at 300 K using 2D and 3D simulations.

Further, applying ML regression models for data prediction represents a key novelty in this study, as they uncover deeper relationships between factors influencing the proposed photodetector’s performance. This research addresses a significant gap in the literature by providing a thorough assessment and comparative analysis of diverse ML regression models applied to the photodetector. The most efficient approach for enhancing both design optimization and predictive modeling is identified through the use of decision trees (DT), random forest (RF), extra trees (ET), and gradient boosting machine (GBM) regression models, which create predictive models capable of estimating critical photodetector electrical and optical parameters based on design and material composition. These models are specifically chosen for their ability to handle complex, non-linear relationships often found in semiconductor device characteristics. Ultimately, they could aid in optimizing the design and fabrication of photodetectors for specific applications, potentially accelerating the development of high-performance devices This approach offers a powerful tool for exploring the parameter space of photodetector design through these ML techniques, potentially revealing insights that might go undetected by traditional analytical methods.

## Parameters of Hg_1–*x*_Cd_*x*_Te

**Table 1:** Parameters of Hg_1–*x*_Cd_*x*_Te as a function of *x* and *T*.

Parameter	Values
Energy bandgap (*E*_g_) (eV)	$\: - {{0}}{{.302 + 5}}{{.35 \times 1}}{{{0}}^{{{ - 4}}}}\left( {\frac{{{{{T}}^{{3}}} - {{1822}}}}{{{{{T}}^{{2}}}\:{{ + }}\:{{255}}.{{2}}}}} \right)\left( {{{1}} - {{2x}}} \right){{ + 1}}{{.93x}} - {{0}}{{.810}}{{{x}}^{{2}}}{{ + 0}}{{.832}}{{{x}}^{{3}}}$
Cut-off wavelength (λ_c_) (µm)	$\:\frac{1.24}{{E}_{\text{g}}\left(e\text{V}\right)}$
Effective mass of the electron ($\:{\varvec{m}}_{\varvec{n}}^{\varvec{*}}$) (kg)	$\:\frac{{{m}}_{\text{0}}}{\left[\text{1+2}{F}\text{+}\frac{{{E}}_{\text{p}}}{\text{3}}\left(\frac{\text{1}}{{{E}}_{\text{g}}\text{+}{\Delta}} \text{+}\frac{\text{2}}{{{E}}_{\text{g}}}\right)\right]}$; where *m*_0_ = 9.1 × 10^–31^ kg; *F* = − 0.8; *E*_p_ = 19 eV; and Δ = 1 eV
Effective mass of hole $\:\left({\varvec{m}}_{\varvec{p}}^{\varvec{*}}\right)$ (kg)	0.55*m*_0_
Intrinsic carrier density (*n*_i_) (cm^–3^)	$$\begin{array}{lll}& ( - 5.77046{{{x}}^2} - 3.57290x + 1.25942 \times {10^{ - 2}}{{xT}}\\& - 4.74019 \times {10^{ - 4}}{{T}} - 4.24123 \times {10^{ - 6}}{{{T}}^2} + 5.24256)\\& \times {10^{14}}{{{E}}_{\text{g}}}^{0.75}{{{T}}^{1.5}}{{\text{e}}^{\left( {\frac{{ - {{q}}{{{E}}_{\text{g}}}}}{{2{k_{\text{B}}}{{T}}}}} \right)}}\end{array}$$
Electron mobility (*µ*_n_) (cm^2^/Vs)	$\:\frac{9\times\:1{0}^{8}S}{{T}^{2r}};\:\text{w}\text{h}\text{e}\text{r}\text{e}\:S={\left(\frac{0.2}{x}\right)}^{7.5}\:\text{a}\text{n}\text{d}\:r={\left(\frac{0.2}{x}\right)}^{0.6}$
Hole mobility (*µ*_p_) (cm^2^/Vs)	$\:440{\left[1+{\left(\frac{p}{1.8\times\:1{0}^{17}}\right)}^{2}\right]}^{-1/4}$, where *p* is hole density in cm^-3^
Electron affinity (χ) (eV)	$\:-\text{0.813}\left({{E}}_{\text{g}}-\text{0.083}\right)\text{+4.23}$
Static dielectric constant (*ε*_s_)	$\:20.5-15.6x+5.7{x}^{2}$
High-frequency dielectric constant (*ε*_∞_)	$\:15.2-15.6x+8.2{x}^{2}$
Optical absorption coefficient *α*(*λ*) (m^–1^)	$$\:\left\{\begin{array}{c}{{\alpha\:}}_{0}{{e}}^{\left({\delta\:}/{{k}}_{\mathbf{B}}{T}\right)\left({{E}}_{\mathbf{p}}-{{E}}_{0}\right)}\begin{array}{c}\begin{array}{c}\end{array}\end{array}{{E}}_{\mathbf{p}}<{{E}}_{\mathbf{g}}\\\:{{\alpha\:}}_{\mathbf{g}}{{e}}^{{\beta\:}\left({{E}}_{\mathbf{p}}-{E}\mathbf{g}\right)}\begin{array}{cc}&\:\:\:\:\:\:\:{{E}}_{\mathbf{p}}>{{E}}_{\mathbf{g}}\end{array}\end{array}\right.$$; where *E*_p_ is photon energy; $\:{\alpha}_{\text{0}}\text{=}{\text{e}}^{\left(-\:\text{18.5}\text{+}45.68{x}\:\right)}$; $\:{{E}}_{\text{0}}\text{=1.77}{x}-\text{0.355}$; $\:\frac{{\delta\:}}{{{k}}_{\mathbf{B}}{T}}=\frac{\text{ln}{{\upalpha\:}}_{\mathbf{g}}-\text{ln}{{\upalpha\:}}_{0}}{{{E}}_{\mathbf{g}}-{{E}}_{0}}$; $\:{\alpha}_{\text{g}}\text{=}-\text{65+1.88}{T}\text{+}\left(\text{8694}-\text{10.314}{T}\right){x}$; and $\:\beta {\:=\:}-\text{1+0.083}{T}\text{+(21}-\text{0.13}{T}\text{)}{x}$
Refractive index (*n*)	$\:\sqrt{{P}+\frac{{Q}}{\left(1-{\left(\frac{{R}}{{\lambda\:}}\right)}^{2}\right)}+{S}{{\lambda\:}}^{2}}$, $$\begin{array}{llll}& {\rm{where}}\:P\: = \:13.173 + {10^{ - 3}}(300 - T) - 9.852x\:\\& + 2.909{x^2},\:Q\: = 0.838 \times {10^{ - 4}}(300 - T) - 0.246x\\& - 0.0961{x^2},\:R\: = \:6.706 + 7 \times {10^{ - 4}}(300 - T) - 14.437x\\& + 8.531{x^2},\:S\: = 1.953 \times {10^{ - 4}} - 0.00128x + 1.853 \times {10^{ - 4}}{x^2}\end{array}$$
Extinction coefficient (*k*)	$\:\frac{{\lambda\:}{\alpha\:}\left({\lambda\:}\right)}{4{\pi\:}}$; where λ is the incident wavelength

The compound semiconductor Hg_1–*x*_Cd_*x*_Te serves as an excellent material for IR photodetectors, owing to its adjustable energy bandgap that can be tuned across the 1–30 μm wavelength range. Numerical simulations are conducted to theoretically characterize the proposed photodetector operating at 77 K. The Cd mole fraction (*x*) in the Hg_1–*x*_Cd_*x*_Te material is set to 0.2217, yielding an energy bandgap corresponding to the LWIR cut-off wavelength of 10.6 μm. Table 1 presents the characteristic parameters of the proposed photodetector, calculated using empirical formulas that depend on Cd composition and temperature (*T*)^8,22^.

## Proposed p^+^-n^–^ LWIR photodetector design

**Fig. 1:**
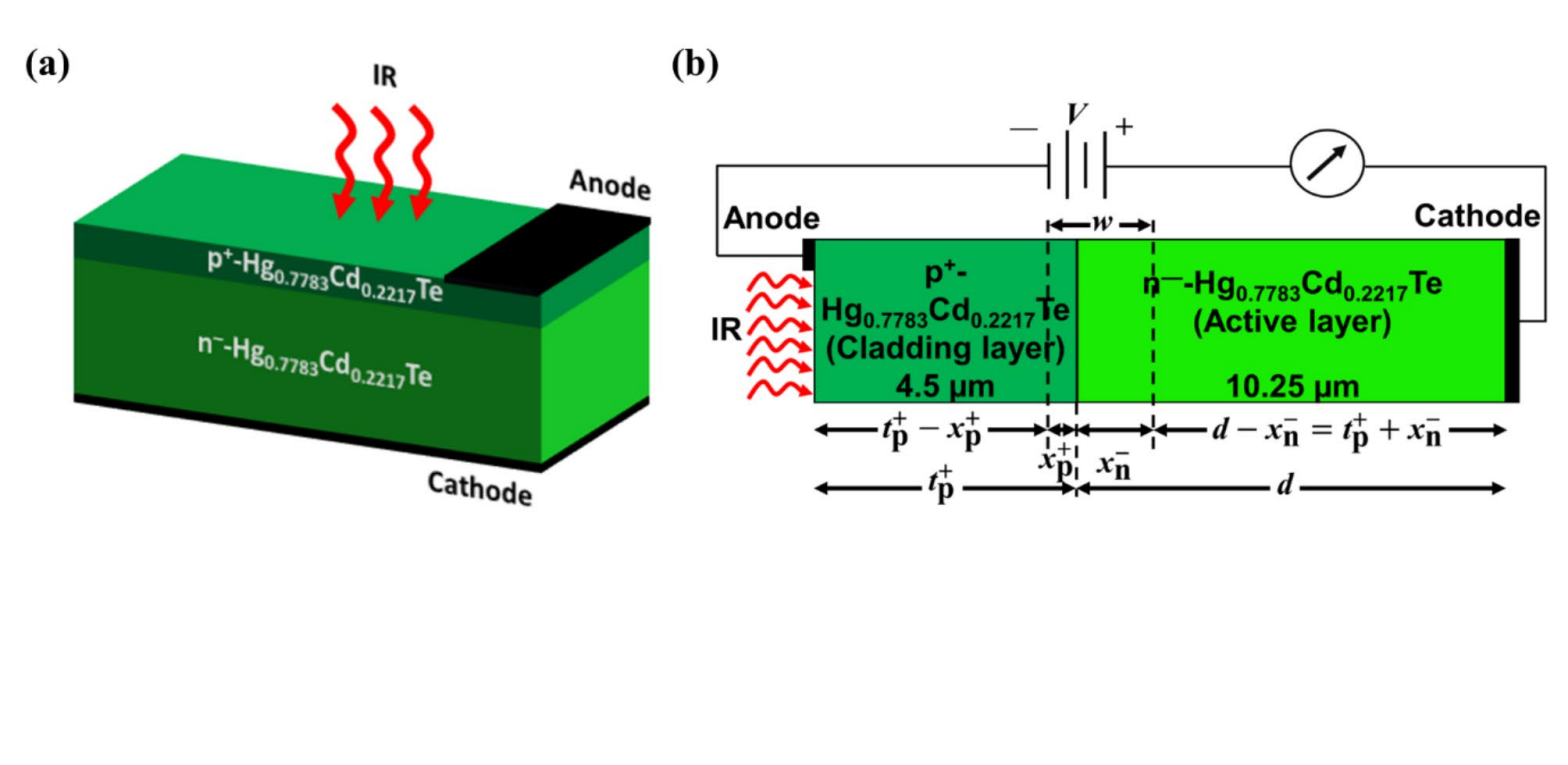
The schematic representation of p^+^-Hg_0.7783_Cd_0.2217_Te/n^–^-Hg_0.7783_Cd_0.2217_Te-based LWIR homojunction photodetector. (**a**) 3D view and (**b**) 2D view. $\:{t}_{\text{p}}^{+}$ and *d* is the thickness of p^+^- and n^–^-regions, respectively. The depletion region width across p^+^- and n^–^-regions is represented by $\:{x}_{\text{p}}^{+}$ and $\:{x}_{\text{n}}^{-}$, respectively. Here *V* is the applied voltage and *w* is the width of the depletion region.

The p^+^–n^–^ LWIR photodetector proposed in Fig. 1 consists of 3D and 2D schematics of two layers of Hg_1–*x*_Cd_*x*_Te: a 4.5 μm thick, heavily doped p^+^ layer serving as the cladding layer, and a 10.25 μm thick, lightly doped n^–^ layer functioning as the active layer. A broader active region allows for increased photon absorption, resulting in enhanced photodetection performance. This structure can be epitaxially grown on a CdZnTe substrate. This research, however, excludes analysis of the substrate’s influence, as it is considered to have minimal impact on the photodetector’s overall functionality. The LWIR operational range of the photodetector is defined by the active layer’s Cd composition, which is set at 0.2217. The same Cd composition is applied to both the cladding and active layers to create a homojunction structure. The photodetector is designed with incident light of 1 W/cm^2^ intensity on the p^+^ layer to ensure sufficient radiation absorption. Both p^+^- and n^–^-regions absorb the LWIR radiation. The photodetector operates under reverse bias, with most of the voltage drop occurring across the lightly doped active layer (Fig. 1). The p^+^-region has an acceptor concentration of 10^17^ cm^–3^, while the n^–^-region has a donor concentration of 10^15^ cm^–3^.

The active region of the photodetector features a strong electric field, which rapidly sweeps photogenerated carriers to their respective electrodes, thereby enhancing operational speed. The photodetector is optimized to maximize the absorption of incident radiation within the active region. This design strategy eliminates the need for photogenerated carriers to diffuse from neutral regions, further enhancing the photodetector’s efficiency and speed.

## Numerical simulation of photodetector

The design and simulation of the p^+^–n^–^ homojunction photodetector is conducted using Silvaco’s ATLAS device simulator platform. The 2D and 3D device structure is carefully simulated in ATLAS to evaluate and analyze the performance parameters of the proposed photodetector. ATLAS employs a finite element method to determine the optoelectronic characteristics of the photodetector. The proposed photodetector’s electrical characteristics are analyzed by solving fundamental semiconductor equations. These include the continuity equations and Poisson’s equation for both electrons and holes, which can be approximated as follows^54^

1 $$\:\frac{\partial\:n}{\partial\:t}=\frac{1}{q}\nabla\:.{J}_{\text{n}}+{G}_{\text{n}}-{R}_{\text{n}}$$

2 $$\:\frac{\partial\:p}{\partial\:t}=-\frac{1}{q}\nabla\:.{J}_{\text{p}}+{G}_{\text{p}}-{R}_{\text{p}}$$

3 $$\:{\nabla\:}^{2}V=-\frac{\rho\:}{\epsilon\:}$$

In these equations, *q* represents the electronic charge, while *n* and *p* denote the equilibrium concentrations of electrons and holes. *J*_n_ and *J*_p_ represent the current densities for electrons and holes, respectively. *G*_n_ and *G*_p_ represent the generation rates for electrons and holes, respectively, while *R*_n_ and *R*_p_ indicate their respective recombination rates, respectively. The electrostatic potential is denoted by *V*, *ρ* represents the space charge density, and *ε* stands for the material’s permittivity. The Silvaco ATLAS device simulator typically solves both continuity equations but can be configured to solve for either electrons or holes individually using a method statement. For simulating carrier transport in semiconductors, the drift-diffusion model is most commonly employed. This model approximates current density equations using Boltzmann transport theory, expressing them as functions of electric field $\:\left(E=-\nabla\:V\right)$ and carrier concentrations (*n* and *p*). The resulting expressions include both drift and diffusion components^54^

4 $$\:{J}_{\text{n}}=q{\mu\:}_{\text{n}}nE+q{D}_{\text{n}}\nabla\:n$$

5 $$\:{J}_{\text{p}}=q{\mu\:}_{\text{p}}pE-q{D}_{\text{p}}\nabla\:p$$

The diffusion coefficients for electrons (*D*_n_) and holes (*D*_p_) are estimated using the following approximations

6 $$\:{D}_{\text{n}}=\frac{{\mu\:}_{\text{n}}{k}_{\text{B}}T}{q}{\text{c}\text{m}}^{2}/\text{s}$$

7 $$\:{D}_{\text{p}}=\frac{{\mu\:}_{\text{p}}{k}_{\text{B}}T}{q}{\text{c}\text{m}}^{2}/\text{s}$$

where *k*_B_ denotes the Boltzmann’s constant.

To analyze the optoelectronic properties, the carrier transport diffusion, Poisson, and continuity equations based on Boltzmann’s transport model are solved with suitable boundary conditions by using Newton’s iterative numerical method. The carrier lifetime and dark current density in the proposed photodetector are characterized by incorporating Shockley–Read–Hall (SRH), Auger, and optical (band-to-band) recombination rates. These rates are expressed as follows^54^

8 $$\:{R}_{\text{SRH}}=\frac{pn-{n}_{\text{i}}^{2}}{{\tau\:}_{\text{p}}\left[n+{n}_{\text{i}}\text{exp}\left({E}_{\text{t}}/{k}_{\text{B}}T\right)\right]+{\tau\:}_{\text{n}}\left[p+{n}_{\text{i}}\text{exp}\left(-{E}_{\text{t}}/{k}_{\text{B}}T\right)\right]}$$

9 $$\:{R}_{\text{Auger}}={C}_{\text{n}}\left(p{n}^{2}-n{n}_{\text{i}}^{2}\right)+{C}_{\text{p}}\left({p}^{2}n-p{n}_{\text{i}}^{2}\right)$$

10 $$\:{R}_{\text{np}}^{\text{Optical}}={C}_{\text{c}}^{\text{OPT}}\left(pn-{n}_{\text{i}}^{2}\right)$$

where *τ*_p_ and *τ*_n_ depict the SRH lifetimes of holes and electrons, respectively. *E*_t_ denotes the energy level of traps within the bandgap. *C*_n_ and *C*_p_ are the Auger coefficient of electrons and holes, respectively, and $\:{C}_{\text{c}}^{\text{OPT}}$ denotes the capture rate of carriers.

The optical characterization is performed using LUMINOUS, an integrated component of the ATLAS tool, which calculates photogeneration at each mesh point in the structure. LUMINOUS employs a ray tracing method, conducting two simultaneous calculations to determine optical intensity using *n* and *k*. This procedure calculates the absorption at every mesh point, producing a photogeneration profile in the photodetector that varies with wavelength.

LUMINOUS does not directly compute optical characteristics such as quantum efficiency, photocurrent responsivity, specific detectivity, and noise equivalent power. Instead, it utilizes the currents obtained from the simulation to derive these optical properties. The simulation employs a monochromatic light source, with the source photocurrent (*I*_S_) and available photocurrent (*I*_A_) expressed as^54^

11 $$\:{I}_{\text{S}}=\frac{q{B}_{\text{n}}\lambda\:}{hc}{W}_{\text{t}}$$

12 $$\:{I}_{\text{A}}=\frac{q{B}_{\text{n}}\lambda\:}{hc}\sum\:_{i=1}^{{N}_{\text{R}}}{W}_{\text{R}}{\int\:}_{0}^{{Y}_{i}}{P}_{\text{i}}{\alpha\:}_{\text{i}}{e}^{-{\alpha\:}_{\text{i}}y}dy$$

where *B*_n_ denotes the intensity of beam number n. The beam width is represented by *W*_t_, while *W*_R_ stands for the ray width. Planck’s constant is symbolized by *h*, and *c* represents the speed of light. The material’s absorption coefficient is denoted by *α*_i_, while *N*_R_ indicates the number of rays traced. *P*_i_ represents the attenuation before the ray’s starting point, and *Y*_*i*_ signifies the ray’s length.

In this context, *I*_S_ denotes the rate of photons incident on the device, while *I*_A_ represents the rate of photons absorbed by the device. Generally, *I*_A_ is lower than *I*_S_ because some of the incident illumination is reflected off the device surface or transmitted through it without being absorbed^22,54^.

### Carrier lifetime modeling

To accurately estimate the dark current density, it is necessary to calculate both the drift and diffusion components precisely. The modeling of the lifetime of minority carriers must be done by considering all possible recombination mechanisms, including radiative recombination, SRH recombination, and Auger recombination. Advancements in fabrication techniques lead to a reduction in SRH recombination. For photodetectors constructed from narrow bandgap materials, Auger and radiative mechanisms predominate. The radiative process occurs when a free electron recombines with a hole, emitting the excess energy as a photon.

#### Radiative recombination mechanism

This process is an inherent characteristic of the material, determined by its electronic band structure. The rate of recombination can be described by the following equation^55^

13 $$\:{G}_{\text{R}}=\frac{8\pi\:}{{h}^{3}{c}^{3}}{\int\:}_{0}^{\infty\:}\frac{\epsilon\:\left(\text{E}\right)\alpha\:\left(\text{E}\right){E}^{2}dE}{{e}^{\left(\frac{E}{{k}_{\text{B}}T}\right)}-1}$$

where *E* represents the electric field across the junction, *ε*(E) denotes the relative dielectric constant, and *α*(E) is the absorption coefficient and is given as^55^

14 $$\:\alpha\:\left(\text{E}\right)=\frac{{2}^{\raisebox{1ex}{$2$}\!\left/\:\!\raisebox{-1ex}{$3$}\right.}{m}_{0}}{3\sqrt{{\epsilon\:}_{\infty\:}}}{\left(\frac{q}{\hslash\:}\right)}^{2}{\left(\frac{{m}_{\text{p}}^{*}{m}_{\text{n}}^{*}}{{m}_{0}\left({m}_{\text{p}}^{*}+{m}_{\text{n}}^{*}\right)}\right)}^{3/2}\left(1+\frac{{m}_{0}\left({m}_{\text{p}}^{*}+{m}_{\text{n}}^{*}\right)}{{m}_{\text{p}}^{*}{m}_{\text{n}}^{*}}\right)\sqrt{\frac{E-{E}_{\text{g}}}{{m}_{0}{c}^{2}}}$$

where $\:\hslash\:=\frac{h}{2\pi\:}$ denotes the reduced Planck’s constant and

15 $$\:E=\left[\frac{2q}{{\epsilon\:}_{0}{\epsilon\:}_{\text{s}}}\left(\frac{{E}_{\text{g}}}{q}\pm\:V\right)\frac{{n}_{0}{p}_{0}}{{n}_{0}+{p}_{0}}\right]$$

where *ε*_0_ is permittivity in vacuum and *V* is the applied bias voltage. *p*_0_ and *n*_0_ represent the thermal-equilibrium concentration of holes and electrons, respectively.

The carrier lifetime resulting from the radiative recombination process can be calculated using the high-frequency dielectric constant *ε*_∞_ and ignoring the dispersion in the dielectric constant. It is expressed as^55^

16 $$\:{\tau\:}_{\text{R}}=\frac{{n}_{\text{i}}^{2}}{{G}_{\text{R}}\left({p}_{0}+{n}_{0}\right)}$$

Assuming that *E*_g_*> k*_B_*T* and neglecting the dispersion in the dielectric constant, the rate of recombination can be rewritten as follows^55^

17 $$\:{G}_{\text{R}}=5.8\times\:{10}^{-13}{n}_{\text{i}}^{2}\sqrt{{\epsilon\:}_{\infty\:}}{\left(\frac{300}{{k}_{\text{B}}T}\right)}^{3/2}\left(1+\frac{{m}_{0}}{{m}_{\text{n}}^{*}}\right){\left(\frac{{m}_{0}}{{m}_{\text{p}}^{*}+{m}_{\text{n}}^{*}}\right)}^{3/2}\left({E}_{\text{g}}^{2}+3{k}_{\text{B}}T{E}_{\text{g}}+3.75{k}_{\text{B}}^{2}{T}^{2}\right)$$

#### Shockley Read Hall (SRH) recombination mechanism

SRH recombination is an extrinsic process that takes place via energy levels within the forbidden bandgap. It isn’t an inherent constraint on photodetector performance and can be minimized by employing higher purity and quality materials, which is feasible for narrow bandgap materials. The carrier lifetime resulting from SRH recombination is represented by the following Eq. 

18 $$\:{\tau\:}_{\text{SRH}}=\frac{1}{\sigma\:{N}_{\text{t}}{v}_{\text{th}}}$$

where *σ* denotes the capture cross-section for minority carriers, *N*_t_ represents the density of SRH traps, and *v*_th_ symbolizes the thermal velocity of minority carriers, which is expressed as

19 $$\:{v}_{\text{th}}=\sqrt{\frac{3{k}_{\text{B}}T}{{m}_{n}^{*}}}$$

#### Auger recombination mechanism

In narrow bandgap semiconductor materials, Auger recombination plays a crucial role. Among the various Auger mechanisms, three stand out as the most significant. In n-type materials, the Auger-1 process is the most common. For p-type materials, Auger-7 is the predominant mechanism. For narrow bandgap materials where the energy bandgap is less than the spin split-off energy, as in this case, the Auger-S process can typically be neglected. Consequently, when dealing with Hg_1–*x*_Cd_*x*_Te material, only the Auger-1 and Auger-7 processes need to be taken into account. Therefore, Auger lifetime (*τ*_Aug_) is given by

20 $$\:\frac{1}{{\tau\:}_{\text{A}\text{u}\text{g}}}=\frac{1}{{\tau\:}_{\text{A}\text{u}\text{g}1}}+\frac{1}{{\tau\:}_{\text{A}\text{u}\text{g}7}}$$

where *τ*_Aug1_ represents the carrier lifetime associated with Auger-1 transitions, while *τ*_Aug7_ denotes the carrier lifetime resulting from Auger-7 transitions^55^.

Taking into account all recombination processes affecting carrier behavior, the overall lifetime can be represented as

21 $$\:\frac{1}{\tau\:}=\frac{1}{{\tau\:}_{\text{R}}}+\frac{1}{{\tau\:}_{\text{S}\text{R}\text{H}}}+\frac{1}{{\tau\:}_{\text{A}\text{u}\text{g}}}$$

## Theoretical modeling of photodetector

### Dark current density

The photodetector’s performance is significantly affected by the total dark current density (*J*_dark_), which results from thermally generated carriers. In modeling the *J*_dark_ of the photodetector, all mechanisms occurring in various regions have been taken into account, except for the current due to the drift mechanism. The components of *J*_dark_ originate from three main sources:

1. diffusion of thermally generated carriers in the neutral region (*J*_diff_),
2. generation-recombination of carriers in the depletion region (*J*_gr_) and.
3. tunneling of carriers across the junction (*J*_Tun_).

The tunneling current density consists of two parts: Band-to-band (BTB) tunneling and Trap-assisted tunneling (TAT), which occurs via defect levels in the material’s bandgap. Consequently, the total dark current density, a function of applied voltage *V* and ambient temperature *T*, can be expressed as^22^

22 $$\:{J}_{\text{dark}}\left(V,T\right)={J}_{\text{diff}}+{J}_{\text{gr}}+{J}_{\text{TAT}}+{J}_{\text{BTB}}$$

where

23 $$J_{\text{diff}}=[\:{\left({\text{J}}_{{\text{p}}^{\text{+}}}\right)}_{{\text{n}}^{-}}+\:{\left({\text{J}}_{{\text{n}}^{-}}\right)}_{{\text{p}}^{\text{+}}}](e^{qV/k_{\text{B}}T}-1)$$

The diffusion contributions to current density for holes in n^–^- regions and electrons in p^+^-regions are denoted by $\:{\left({\text{J}}_{{\text{p}}^{\text{+}}}\right)}_{{\text{n}}^{-}}$ and $\:{\left({\text{J}}_{{\text{n}}^{-}}\right)}_{{\text{p}}^{\text{+}}}$, respectively. These components are estimated using the following approximations

24 $$\:{\left({J}_{{\text{p}}^{+}}\right)}_{{\text{n}}^{-}}=\frac{q{n}_{\text{i}}^{2}}{{N}_{\text{D}}}\sqrt{\frac{{\mu\:}_{\text{p}}{k}_{\text{B}}T}{q{\tau\:}_{\text{p}}}}\frac{\frac{{S}_{\text{p}}{L}_{\text{p}}}{{D}_{\text{p}}}\text{cosh}\left(\frac{d-{x}_{\text{n}}^{-}}{{L}_{\text{p}}}\right)+\text{sinh}\left(\frac{d-{x}_{\text{n}}^{-}}{{L}_{\text{p}}}\right)}{\text{cosh}\left(\frac{d-{x}_{\text{n}}^{-}}{{L}_{\text{p}}}\right)+\frac{{S}_{\text{p}}{L}_{\text{p}}}{{D}_{\text{p}}}\text{sinh}\left(\frac{d-{x}_{\text{n}}^{-}}{{L}_{\text{p}}}\right)}$$

25 $$\:{\left({J}_{{\text{n}}^{-}}\right)}_{{\text{p}}^{+}}=\frac{q{n}_{\text{i}}^{2}}{{N}_{\text{A}}}\sqrt{\frac{{\mu\:}_{\text{n}}{k}_{\text{B}}T}{q{\tau\:}_{\text{n}}}}\frac{\frac{{S}_{\text{n}}{L}_{\text{n}}}{{D}_{\text{n}}}\text{cosh}\left(\frac{{t}_{\text{p}}^{+}-{x}_{\text{p}}^{+}}{{L}_{\text{n}}}\right)+\text{sinh}\left(\frac{{t}_{\text{p}}^{+}-{x}_{\text{p}}^{+}}{{L}_{\text{n}}}\right)}{\text{cosh}\left(\frac{{t}_{\text{p}}^{+}-{x}_{\text{p}}^{+}}{{L}_{\text{n}}}\right)+\frac{{S}_{\text{n}}{L}_{\text{n}}}{{D}_{\text{n}}}\text{sinh}\left(\frac{{t}_{\text{p}}^{+}-{x}_{\text{p}}^{+}}{{L}_{\text{n}}}\right)}$$

where *N*_D_ and *N*_A_ are the donor and acceptor concentrations, respectively. *τ*_p_ and *τ*_n_ correspond to the hole and electron lifetimes, respectively. *S*_p_ and *S*_n_ are the surface recombination velocities of holes and electrons, respectively. $\:{L}_{\text{p}}=\sqrt{{D}_{\text{p}}{\tau\:}_{\text{p}}}$ and $\:{L}_{\text{n}}=\sqrt{{D}_{\text{n}}{\tau\:}_{\text{n}}}$ are the diffusion lengths of holes and electrons, respectively, in cm.

The movement of charge carriers across the p^+^-n^–^ junction is significantly influenced by traps or defects within the depletion region. The current density components for electrons and holes, resulting from generation-recombination processes in the depletion region, are expressed as^55^

26 $$\:{J}_{\text{gr}}=\left\{\begin{array}{c}\frac{q{n}_{\text{i}}wV}{\left({V}_{\text{bi}}-V\right){\tau\:}_{\text{SRH}}}\begin{array}{ccccc}&\:&\:&\:&\:V<0\end{array}\\\:\frac{2{n}_{\text{i}}w{k}_{\text{B}}T}{\left({V}_{\text{bi}}-V\right){\tau\:}_{\text{SRH}}}\text{sinh}\left(\frac{qV}{2{k}_{\text{B}}T}\right)\begin{array}{cc}&\:\:\:V>0\end{array}\end{array}\right.$$

where *V*_bi_ is the built-in potential.

Trap-assisted tunneling current density is commonly observed in devices under low reverse bias conditions. This phenomenon occurs at the depletion region boundaries, where minority carriers traverse to unoccupied states on the junction’s opposite side. TAT involves a two-stage process: initially, electrons move from the valence band to a trap state within the bandgap, then tunnel to the conduction band. These intermediate energy levels, acting as trap states, are formed by impurities present in the material.

For materials with a narrow bandgap, the current density resulting from band-to-band tunneling becomes prominent when a high reverse bias is applied. However, this component is less relevant for the proposed photodetector, which typically operates at relatively lower reverse bias voltages.

The TAT component of the current density can be analytically evaluated using the following expression^56^

27 $$\:{J}_{\text{TAT}}=\frac{2{\pi\:}^{2}{q}^{2}{m}_{\text{n}}^{*}W{{}_{\text{c}}{}^{2}N}_{\text{t}}\left({V}_{\text{bi}}-V\right)w}{{h}^{3}\left({E}_{\text{g}}-{E}_{\text{t}}\right)}{e}^{\left(-\frac{\sqrt{3}w{E}_{\text{g}}^{2}}{8\sqrt{2}P\left({V}_{\text{bi}}-V\right)}\alpha\:\left(\frac{{E}_{t}}{{E}_{\text{g}}}\right)\right)}$$

where

28 $$\:\alpha\:\left(\frac{{E}_{\text{t}}}{{E}_{\text{g}}}\right)=\frac{\pi\:}{2}+\text{si}{\text{n}}^{-1}\left(\pm\:1\pm\:2\frac{{E}_{\text{t}}}{{E}_{\text{g}}}\right)\pm\:\left(1-2\frac{{E}_{\text{t}}}{{E}_{\text{g}}}\right)\sqrt{\frac{{E}_{\text{t}}}{{E}_{\text{g}}}\left(1-\frac{{E}_{\text{t}}}{{E}_{\text{g}}}\right)}$$

In this equation, *W*_c_ represents the matrix element related to the trap potential, while *P* denotes the interband matrix element. *E*_t_ signifies the energy position of trap levels within the bandgap.

The current density component due to the BTB tunneling mechanism is approximated by^56^

29 $$\:{J}_{\text{BTB}}=\frac{{q}^{3}EV}{4{\pi\:}^{2}{\hslash\:}^{2}}\sqrt{\frac{2{m}_{n}^{*}}{{E}_{\text{g}}}}{e}^{\left(-\frac{4\sqrt{2{m}_{n}^{*}{E}_{\text{g}}^{3}}}{3q\hslash\:E}\right)}$$

### Resistance-area product

The various current density components discussed earlier contribute to the net resistance-area product (*RA*)_NET_, which can be expressed as

30 $$\:\frac{1}{{\left(RA\right)}_{\text{NET}}}=\frac{1}{{\left(RA\right)}_{\text{diff}}}+\frac{1}{{\left(RA\right)}_{\text{gr}}}+\frac{1}{{\left(RA\right)}_{\text{TAT}}}+\frac{1}{{\left(RA\right)}_{\text{BTB}}}$$

where $\:{\left(RA\right)}_{i}={\left(\frac{d{\left({J}_{\text{d}\text{a}\text{r}\text{k}}\right)}_{i}}{dV}\right)}^{-1}$. The subscript *i* denotes the various components that make up *J*_dark_.

### Photocurrent density

When the photodetector is exposed to light, it generates a photocurrent density. This photocurrent density is influenced by both the applied voltage and the temperature. It can be mathematically represented as^29,57^

31 $$\:{J}_{\text{light}}\left(V,T\right)={J}_{\text{dark}}\left(V,T\right)-\frac{QE{\lambda\:}_{c}{P}_{\text{in}}}{1.24}$$

where the latter term signifies the current density generated by incident photons. *QE* is quantum efficiency, *P*_in_ is incident light power density, and λ_c_ is the cut-off wavelength in µm.

### Quantum efficiency

The photodetector’s quantum efficiency *QE* varies with wavelength and acts as a gain factor. The rate at which electron-hole pairs are optically generated can be expressed as a function of the distance *x* from the surface. This relationship can be mathematically represented as^56^

32 $$\:G\left(x\right)=\frac{\alpha\:\left(\lambda\:\right)\left(1-R\right){P}_{\text{i}\text{n}}}{Ahf}{e}^{-x\alpha\:\left(\lambda\:\right)}$$

where *A* denotes the active area of the photodetector, and *f* is the frequency. In general, the Fresnel reflection coefficient *R* at the junction is evaluated by the formula,

33 $$\:R=\frac{{\left({n}_{\text{t}}-{n}_{\text{e}}\right)}^{2}+{k}^{2}}{{\left({n}_{\text{t}}+{n}_{\text{e}}\right)}^{2}+{k}^{2}}$$

where *n*_e_ and *n*_t_ correspond to the refractive index values of the entrance and transmitted medium, respectively. *k* is the extinction coefficient. When *k* is negligibly small, the reflection coefficient can be computed as^28^

34 $$\:R={\left(\frac{{n}_{\text{t}}-{n}_{\text{e}}}{{n}_{\text{t}}+{n}_{\text{e}}}\right)}^{2}$$

The overall *QE* of the photodetector results from contributions from three distinct regions: the neutral p^+^-region, the neutral n^–^-region, and the depletion region^22,23^. Each of these regions plays a role in determining the total *QE* of the proposed homojunction photodetector.

35 $$\:QE={\left(QE\right)}_{{\text{p}}^{+}}+{\left(QE\right)}_{{\text{n}}^{-}}+{\left(QE\right)}_{\text{dep}}$$

where

36 $$\:{\left(QE\right)}_{{\text{p}}^{+}}=\frac{\left(1-R\right)\alpha\:{L}_{\text{n}}}{{\alpha\:}^{2}{L}_{\text{n}}^{2}-1}{e}^{-\left(\alpha\:{t}_{\text{p}}^{+}+\alpha\:{x}_{\text{n}}^{-}\right)}\left[\frac{\left(\frac{{S}_{\text{n}}{L}_{\text{n}}}{{D}_{\text{n}}}-\alpha\:{L}_{\text{n}}\right){e}^{-\alpha\:\left(d-{x}_{\text{n}}^{-}\right)}-\left\{\frac{{S}_{\text{n}}{L}_{\text{n}}}{{D}_{\text{n}}}\text{c}\text{o}\text{s}\text{h}\left(\frac{d-{x}_{\text{n}}^{-}}{{L}_{\text{n}}}\right)+\text{s}\text{i}\text{n}\text{h}\left(\frac{d-{x}_{\text{n}}^{-}}{{L}_{\text{n}}}\right)\right\}}{\text{c}\text{o}\text{s}\text{h}\left(\frac{d-{x}_{\text{n}}^{-}}{{L}_{\text{n}}}\right)+\frac{{S}_{\text{n}}{L}_{\text{n}}}{{D}_{\text{n}}}\text{s}\text{i}\text{n}\text{h}\left(\frac{d-{x}_{\text{n}}^{-}}{{L}_{\text{n}}}\right)}+\alpha\:{L}_{\text{n}}\right]$$

37 $$\:{\left(QE\right)}_{{\text{n}}^{-}}=\frac{\left(1-R\right)\alpha\:{L}_{\text{p}}}{{\alpha\:}^{2}{L}_{\text{p}}^{2}-1}\left[\frac{\left(\alpha\:{L}_{\text{p}}+\frac{{S}_{\text{p}}{L}_{\text{p}}}{{D}_{\text{p}}}\right)-{e}^{-\alpha\:{x}_{\text{p}}^{+}}\left\{\frac{{S}_{\text{p}}{L}_{\text{p}}}{{D}_{\text{p}}}\text{cosh}\left(\frac{{x}_{\text{p}}^{+}}{{L}_{\text{p}}}\right)+\text{sinh}\left(\frac{{x}_{\text{p}}^{+}}{{L}_{\text{p}}}\right)\right\}}{\text{cosh}\left(\frac{{x}_{\text{p}}^{+}}{{L}_{\text{p}}}\right)+\frac{{S}_{\text{p}}{L}_{\text{p}}}{{D}_{\text{p}}}\text{sinh}\left(\frac{{x}_{\text{p}}^{+}}{{L}_{\text{p}}}\right)}-\alpha\:{L}_{\text{p}}{e}^{-\alpha\:{x}_{\text{p}}^{+}}\right]$$

and

38 $$\:{\left(\text{QE}\right)}_{\text{dep}}=\left(\text{1}-\text{R}\right)\left[{e}^{-\alpha\:{\text{x}}_{\text{p}}^{\text{+}}}-{e}^{-\alpha\:\left({\text{t}}_{\text{p}}^{\text{+}}+{\text{x}}_{\text{n}}^{-}\right)}\right]$$

### Photocurrent responsivity

The photodetector’s photocurrent responsivity (*R*_i_) is the ratio of photocurrent density to the incident light power density and is also related to *QE* by.

39 $$\:{R}_{\text{i}}=\frac{{J}_{\text{l}\text{i}\text{g}\text{h}\text{t}}}{{P}_{\text{i}\text{n}}}=QE\left(\frac{q\lambda\:}{hc}\right)\begin{array}{cc}=QE\left(\frac{\lambda\:}{1.24}\right)& \:\end{array} {\text{A/W}}$$

### Specific detectivity

The specific detectivity (D^*^) quantifies a photodetector’s ability to detect minimal signals and is determined using the following equation^22^.

40 $$\:{D}^{*}=\frac{{R}_{\text{i}}}{2}\sqrt{\frac{{\left({R}_{0}A\right)}_{\text{NET}}}{{k}_{\text{B}}T}}                   {\text{cmHz}}^{1/2}/{\text{W}}$$

where $\:{\left({R}_{0}A\right)}_{\text{NET}}={\left(\frac{d{J}_{\text{d}\text{a}\text{r}\text{k}}}{dV}\right)}_{V=0}^{-1}$ is the resistance-area product under the self-powered mode, i.e. at zero bias. A large value of (*R*_0_*A*)_NET_ is preferable as it leads to enhanced specific detectivity.

### Noise equivalent power

The noise equivalent power (*NEP*) represents the weakest optical signal a photodetector can distinguish from its inherent noise and is calculated using the following relation^22^.

41 $$\:NEP=\frac{\sqrt{\varDelta\:fA}}{{D}^{*}} {\text{W/Hz}}^{1/2}$$

where Δ*f* is the bandwidth.

## Silvaco simulation results and discussions

The Silvaco ATLAS TCAD tool has been used to simulate and analyze the photodetector’s electrical and optical characteristics. The ATLAS framework utilizes BLAZE for electrical characterization and LUMINOUS for optical analysis. Uniform doping has been assumed for p^+^- and n^–^-regions. The simulation employs the ANALYTIC model for mobility calculations and incorporates SRH, AUGER, and OPTICAL recombination mechanism models for current density analysis. Standard band-to-band tunneling has also been considered. The DECKBUILD code integrated with ATLAS includes a collection of parameter values. The ATLAS simulation and analytical model account for surface recombination at the homojunction interface and contacts. Dark current density carrier modeling has been performed separately in MATLAB, with the resulting carrier lifetimes integrated into the ATLAS numerical simulation. A separate MATLAB program has been developed to compute absorption coefficients using Chu’s empirical relation^58,59^. This data has been used to create a file containing *n* and *k* values for various wavelengths. For optical characterization, this (*n*, *k*) data file has been incorporated into the DECKBUILD code for ATLAS simulation.

The SRH carrier lifetime of the photodetector is determined to be 0.29 µs. Calculations reveal the effective density of states for electrons in the conduction band (*N*_CB_) to be 2.65 × 10^15^ cm^–3^, while the effective density of states for holes in the valence band (*N*_VB_) is found to be 1.33 × 10^18^ cm^–3^.

**Fig. 2:**
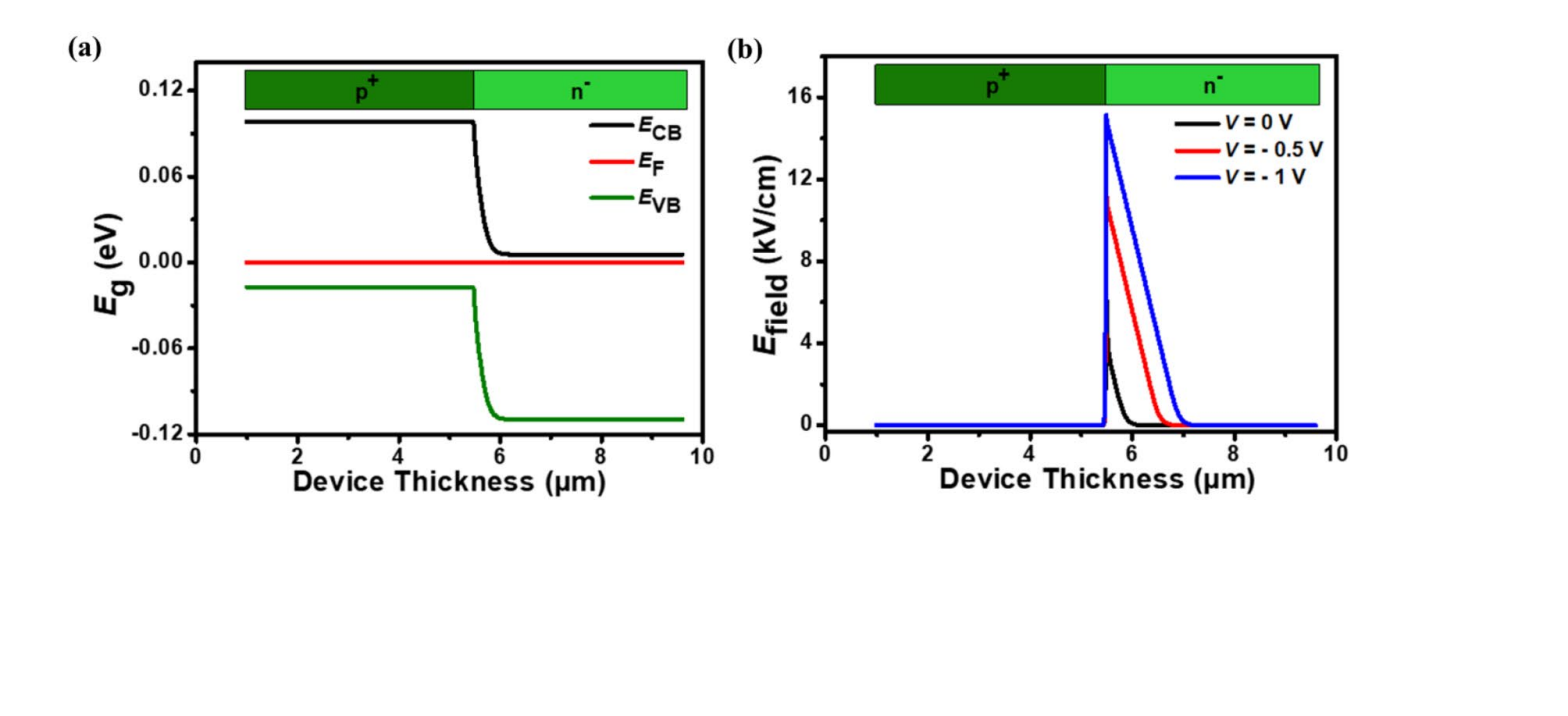
(**a**) Energy bandgap diagram showing the conduction band (*E*_CB_), valence band (*E*_VB_), and Fermi level (*E*_F_) and (**b**) Corresponding electric field intensity profile across the p^+^-Hg_0.7783_Cd_0.2217_Te/n^–^-Hg_0.7783_Cd_0.2217_Te LWIR homojunction photodetector.

Figure 2a presents the energy band diagram of the proposed p⁺–n⁻ homojunction photodetector at 0 V bias under dark conditions. The electric field (*E*_field_) distribution across the photodetector’s junction under dark conditions at different bias voltages is shown in Fig. 2b. The *E*_field_ displays a triangular profile at the interface, reaching maximum values of 7.36, 11.65, and 15.15 kV/cm at 0, − 0.5, and − 1 V bias, respectively.

**Fig. 3:**
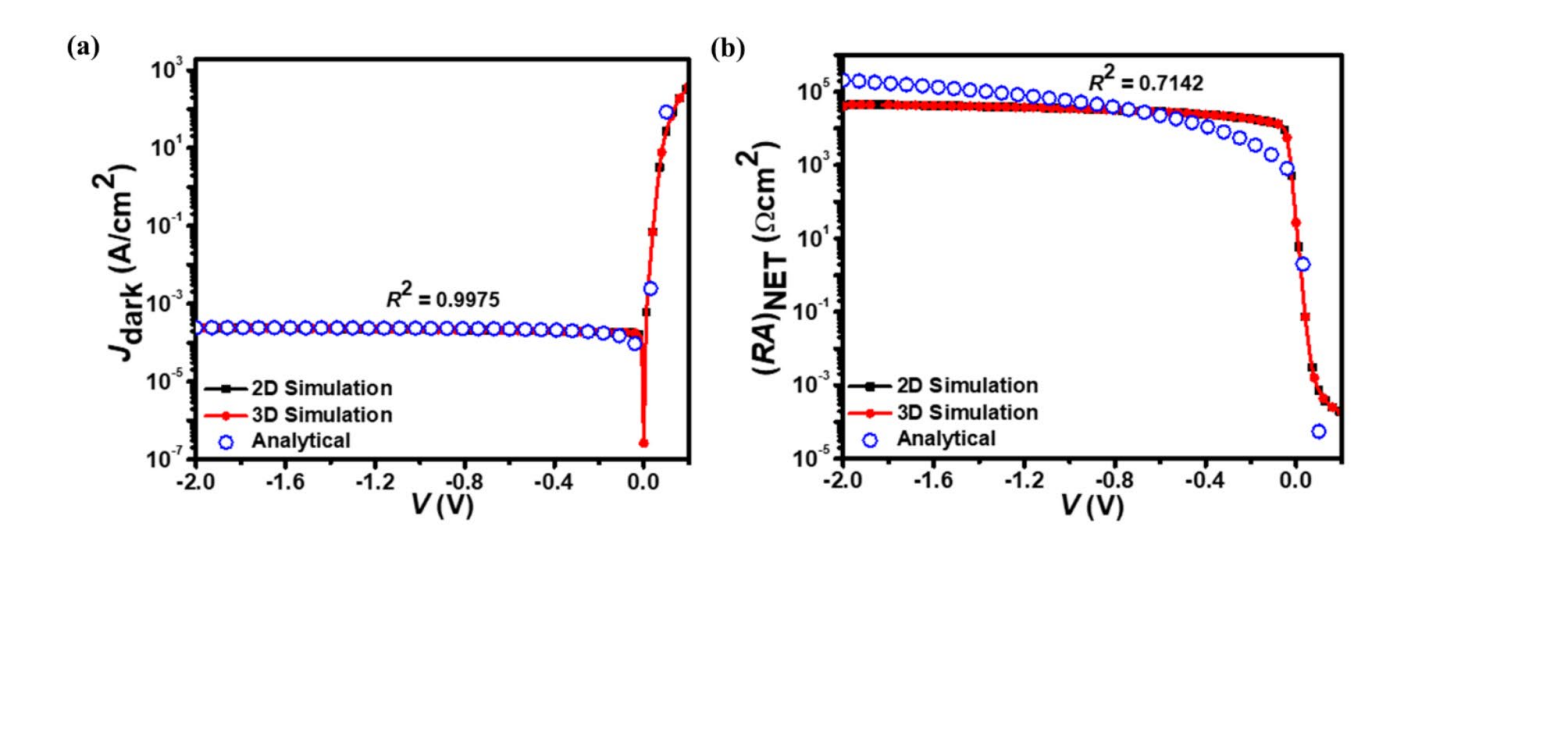
(**a**) The *J*_dark_–*V* and (**b**) (*RA*)_NET_–*V* characteristics of the p^+^-Hg_0.7783_Cd_0.2217_Te/n^–^-Hg_0.7783_Cd_0.2217_Te LWIR homojunction photodetector. The simulated data from both 2D and 3D simulations closely matches the analytical model results (open circles).

Figure 3a illustrates the relationship between *J*_dark_ and *V* in the proposed homojunction photodetector, while Fig. 3b provides the relationship between (*RA*)_NET_ and *V*. In Fig. 3a, the *J*_dark_ increases with reverse bias, which is a characteristic behavior of Hg_1–*x*_Cd_*x*_Te-based photodetectors. This rise in *J*_dark_ is primarily due to the increased carrier generation and tunneling effects at higher reverse biases, leading to enhanced leakage currents. The low *J*_dark_ values observed at zero bias indicate the device’s potential for low-noise operation, which is critical for high-sensitivity IR applications.

The (*RA*)_NET_ value is a critical parameter in assessing the photodetector’s noise performance, where higher values are indicative of lower dark current and improved detectivity. As reverse bias is applied, the (*RA*)_NET_ increases significantly due to the widening of the depletion region. This widening occurs as the majority carriers are pulled away from the junction, resulting in fewer charge carriers available for conduction and thus higher resistance. The increase in (*RA*)_NET_ indicates enhanced resistance to leakage currents, which is essential for maintaining low noise levels in photodetector applications. This behavior emphasizes the critical role of reverse biasing in enhancing the overall efficiency of the photodetector. Furthermore, optimizing (*RA*)_NET_ is crucial for achieving superior specific detectivity and noise equivalent power, thereby enhancing the photodetector’s suitability for high-sensitivity IR sensing. Both graphs demonstrate a strong agreement between the *J*_dark_ and (*RA*)_NET_ values obtained from the 2D and 3D ATLAS simulations and those calculated using the analytical model. This close correlation across different voltage conditions validates the consistency and accuracy of all three approaches in predicting the photodetector’s electrical behavior.

**Fig. 4:**
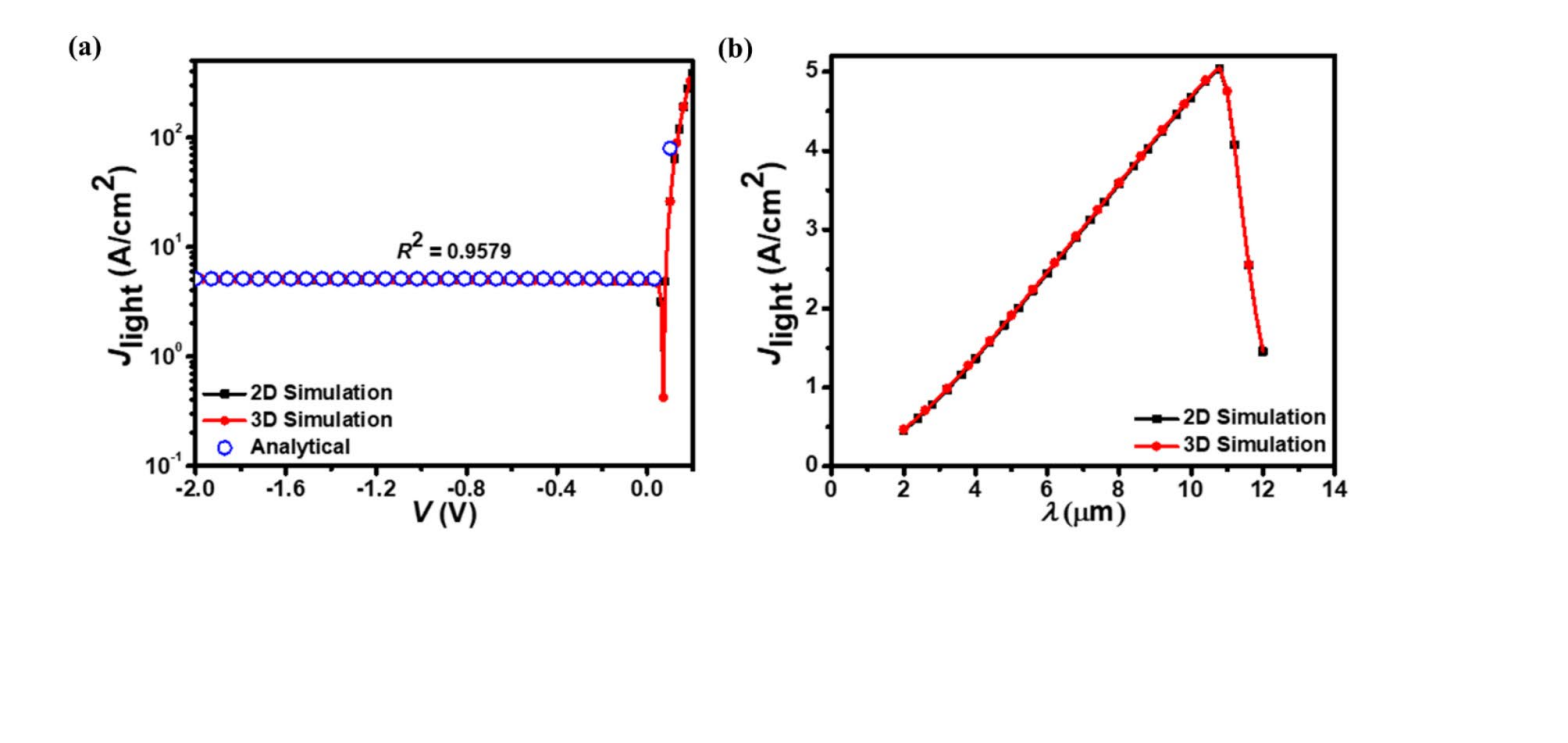
(**a**) The *J*_light_–*V* and (**b**) *J*_light_–*λ* characteristics of the p^+^-Hg_0.7783_Cd_0.2217_Te/n^–^-Hg_0.7783_Cd_0.2217_Te LWIR homojunction photodetector. The *J*_light_-*V* data from both 2D and 3D simulations closely matches the analytical model results (open circles).

Figure 4a, b illustrate the proposed homojunction photodetector’s *J*_light_–*V* and *J*_light_–*λ* characteristics, respectively. The *J*_light_–*λ* curve, plotted at a − 0.5 V bias, shows a rapid decrease in *J*_light_ beyond the 10.6 μm cut-off wavelength, confirming the photodetector’s operation in the LWIR regime. The *J*_light_–*V* curve demonstrates an increase in photocurrent density with increasing voltage, highlighting the voltage-dependent nature of the photodetector’s response.

**Fig. 5:**
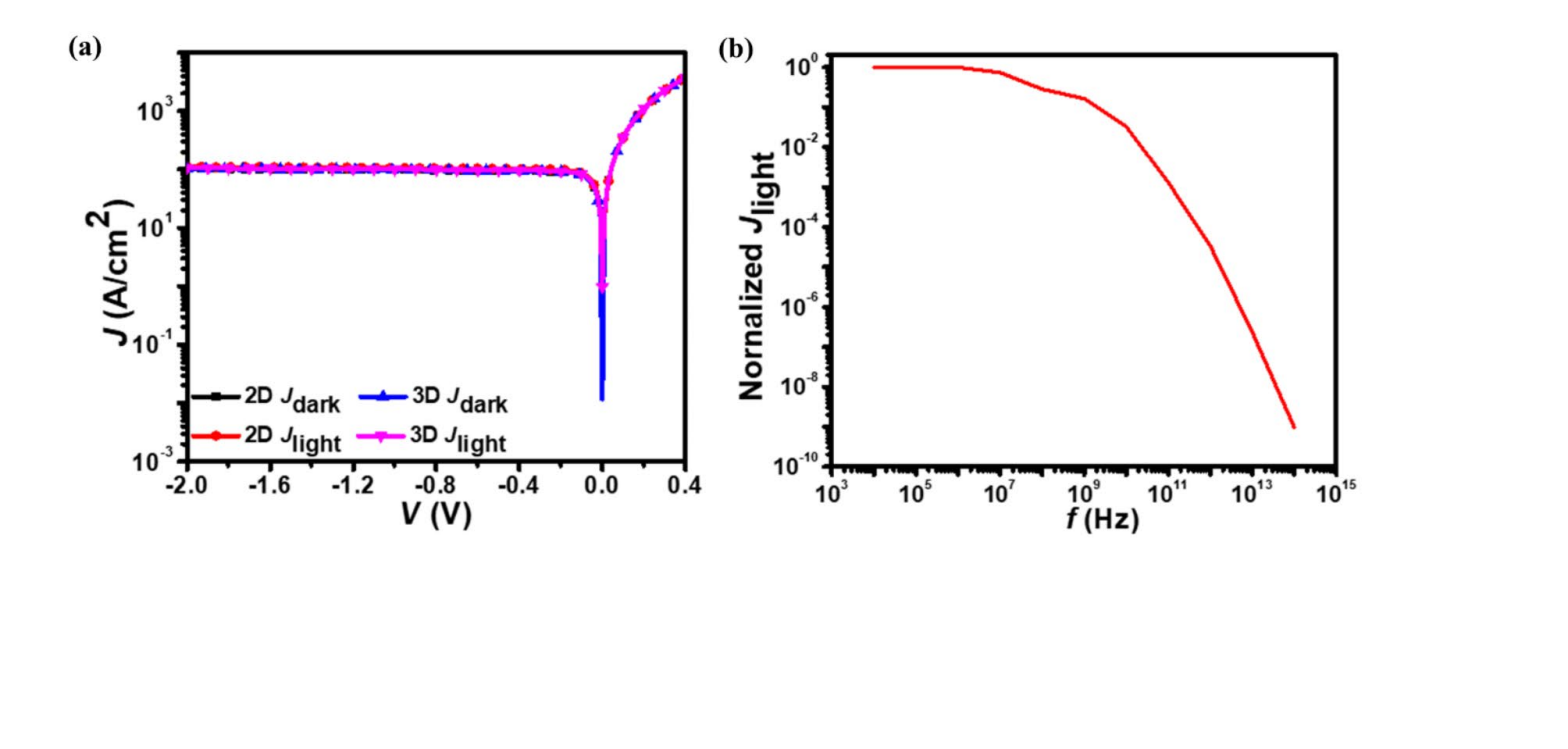
(**a**) The simulated 2D and 3D *J*–*V* characteristics of the p^+^-Hg_0.7783_Cd_0.2217_Te/n^–^-Hg_0.7783_Cd_0.2217_Te LWIR homojunction photodetector at 300 K and (**b**) Normalized frequency response of the p^+^-Hg_0.7783_Cd_0.2217_Te/n^–^-Hg_0.7783_Cd_0.2217_Te LWIR homojunction photodetector at − 0.5 V and 77 K.

The simulated *J*_dark_–*V*, *J*_light_–*V*, and (*RA*)_NET_–*V* characteristics align well with the analytical results, showing coefficient of correlation (*R*^2^) values between 0.7142 and 0.9975. At zero bias, the photodetector demonstrates favorable performance metrics: a low *J*_dark_ of 0.26 µA/cm^2^, a *J*_light_ of 4.9 A/cm^2^, a high *J*_light_/*J*_dark_ ratio of 1.9 × 10^7^, and (*R*_0_*A*)_NET_ of 26.88 Ωcm^2^. The low *J*_dark_ at zero bias is attributed to the photodetector’s inherent potential barrier. When biased at − 0.5 V, the photodetector exhibits a *J*_dark_ of 0.20 mA/cm^2^, a *J*_light_ of 4.98 A/cm^2^, and a *J*_light_/*J*_dark_ ratio of 2.46 × 10^4^. The similarity in results suggests that the 2D and 3D simulations, as well as the analytical method, provide reliable representations of the photodetector’s performance under varying voltage inputs. The inclusion of both 2D and 3D simulations offers a comprehensive view of the photodetector’s characteristics, potentially capturing any dimensionality-dependent effects while also confirming the robustness of the simpler 2D model for this particular analysis.

Figure 5a presents the current density (*J*)–*V* characteristic curves at 300 K under both dark and illumination conditions, derived from 2D and 3D simulations of the proposed photodetector. The results highlight a significant challenge for the room temperature operation of Hg_1–*x*_Cd_*x*_Te-based IR photodetectors. At 300 K, thermal excitation generates substantial dark current density, which often equals or exceeds the photocurrent density^18,22^. This high dark current density severely limits the viability of most Hg_1–*x*_Cd_*x*_Te-based IR photodetectors for practical applications at room temperature.

The normalized frequency response of the proposed photodetector at 77 K and − 0.5 V bias is depicted in Fig. 5b. The photodetector exhibits a 3-dB cut-off frequency (*f*_3 − dB_) of 104 GHz, indicating its potential for use in high-frequency applications.

**Fig. 6:**
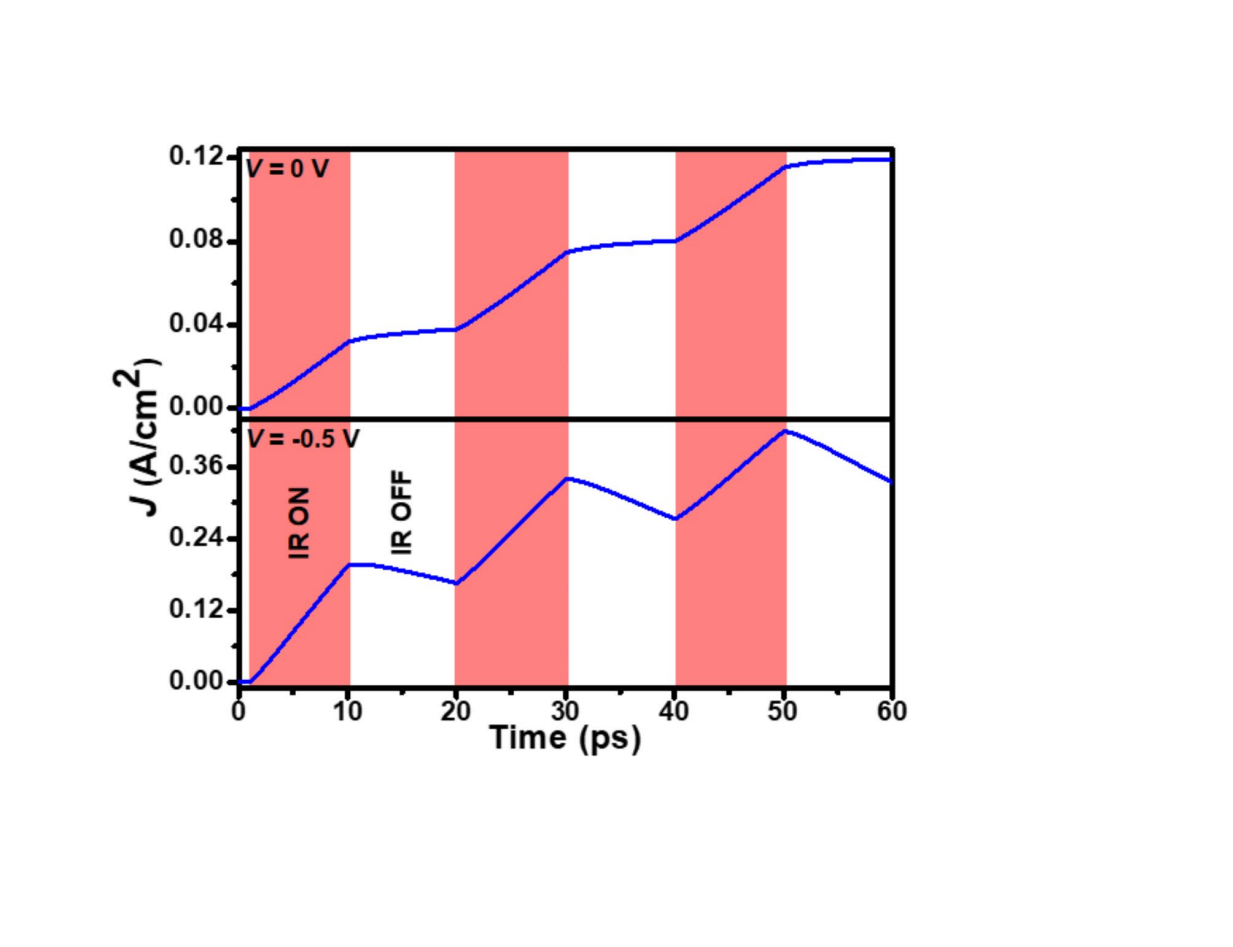
Time-dependent photoresponse of the p^+^-Hg_0.7783_Cd_0.2217_Te/n^–^-Hg_0.7783_Cd_0.2217_Te LWIR homojunction photodetector at 0 V and − 0.5 V.

The photoswitching speed of the proposed photodetector is evaluated by examining its *J*-Time characteristics under 1 W/cm^2^ light illumination at 77 K. Figure 6 illustrates these characteristics at 0 and − 0.5 V bias conditions. The experiment involves pulsing the LWIR radiation in 10 ps intervals and measuring the resulting current density over time. The results reveal persistent photoswitching behavior, characterized by gradual exponential increases and decreases in current during illumination and dark periods, respectively. This figure demonstrates the photodetector’s rapid response to LWIR illumination and its varying sensitivity under different bias conditions. At 0 V, the photodetector exhibits a rise time of 1.62 ps and an instantaneous fall time. When biased at − 0.5 V, the rise time decreases to 0.8 ps, while the fall time increases to 1.2 ps. This behavior can be attributed to the increased electric field strength under reverse bias, which accelerates carrier collection, reducing rise time. The emergence of a measurable fall time at − 0.5 V likely results from the widened depletion region, influencing the carrier sweep-out process upon illumination termination.

The proposed photodetector operates on a timescale dramatically shorter than thermal detectors like bolometers. While bolometers typically respond in milliseconds, the proposed photodetector has a time constant measured in picoseconds (rise time of 1.62 ps at 0 V and 0.8 ps at − 0.5 V). This makes the photodetector approximately nine orders of magnitude faster than thermal detectors^14^. In comparison with other widely used technologies, such as photodiodes or avalanche photodetectors (APDs), the proposed photodetector offers superior speed. Many commercial photodiodes exhibit response times on the order of microseconds or nanoseconds. For example, InGaAs/GaAsSb^34^, InGaAs/InP^35^, InAs/InAsSb^36^, and HgCdTe/black phosphorus van der Waals heterojunction^41^-based photodetectors typically have response times of 6.1 µs, 115 ns, 0.52 ns, and 150 µs respectively, which are significantly slower than the picosecond-scale response achieved in this work. Additionally, APDs, while fast, often require high bias voltages and suffer from excess noise, limiting their performance in low-noise applications^26^. These metrics suggest the proposed photodetector is ideal for applications requiring extremely fast response times, such as high-speed optical switching in telecommunications and optical computing. This speed advantage could enable faster data transmission and signal processing compared to existing technologies. The tunable response times with varying bias also enhance its adaptability to a range of high-speed optical systems. The rapid response and scalability of wearable or flexible electronics make the device a strong candidate for integration into low-power, compact IR sensing systems for biomedical monitoring or Internet of Things (IoT) devices.

**Fig. 7:**
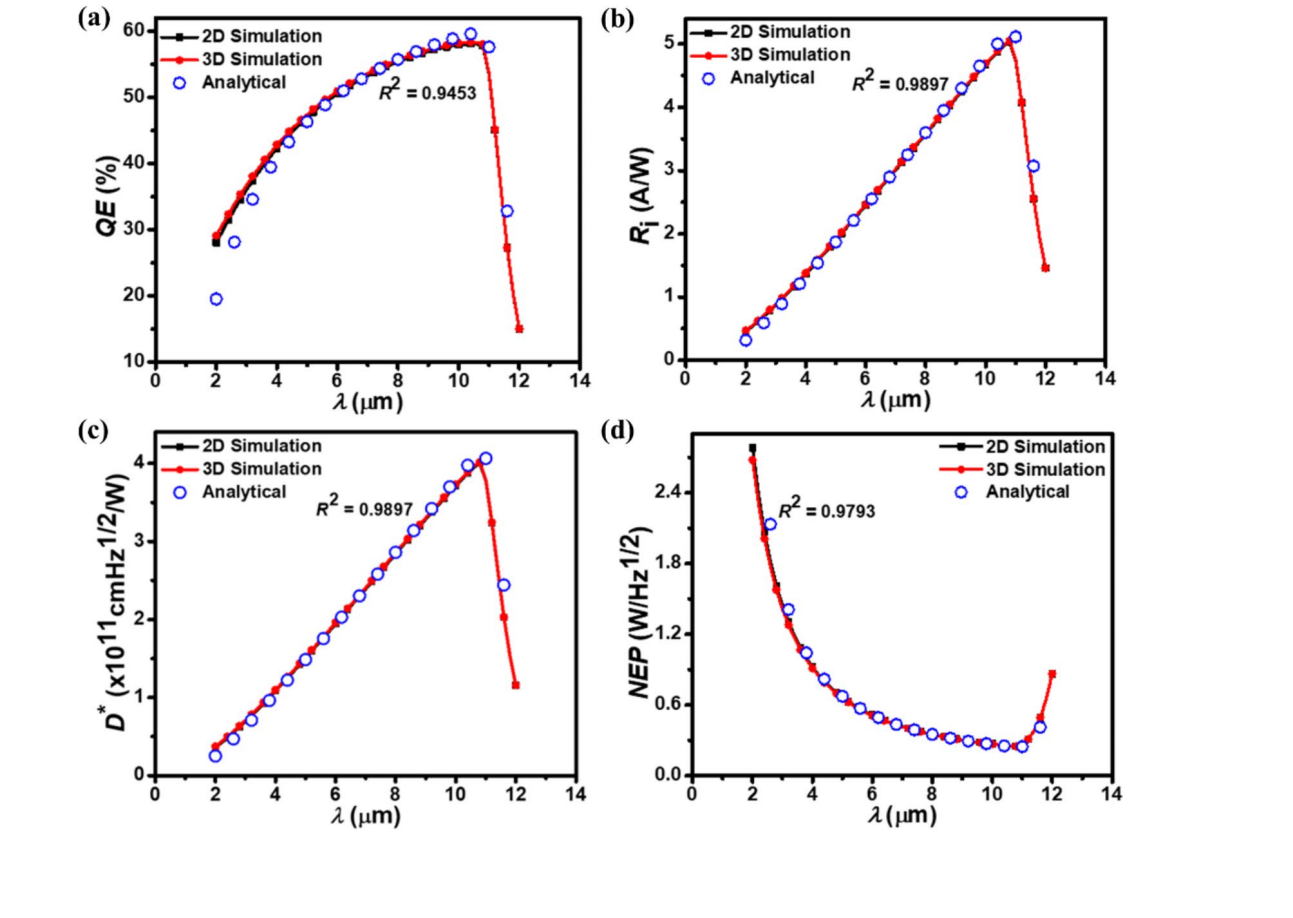
The simulated 2D and 3D optical characteristic parameters of the p^+^-Hg_0.7783_Cd_0.2217_Te/n^–^-Hg_0.7783_Cd_0.2217_Te LWIR homojunction photodetector at − 0.5 V. (**a**) *QE*, (**b**) *R*_i_, (**c**) D^*^, and (**d**) *NEP*. The optical characteristic parameters from both 2D and 3D simulations closely match the analytical model results (open circles).

Figure 7 depicts how optical parameters (*QE*, *R*_i_, D*, and *NEP*) of the photodetector vary with wavelength at − 0.5 V, comparing results from 2D, 3D, and analytical models. The photodetector’s expanded active layer enables greater photon absorption, yielding a *QE* of approximately 58.30 % (Fig. 7a). Beyond the cut-off wavelength, *QE*, *R*_i_, and D* decrease sharply due to increased photon absorption in neutral regions, which limits the efficiency of photon-to-current conversion. This rapid falloff beyond the cut-off wavelength as shown in Fig. 7a–c underscores the photodetector’s wavelength sensitivity, reflecting the design choices in optimizing the active layer thickness and material composition. The figure demonstrates strong agreement between ATLAS 2D and 3D simulation results and those derived from the analytical model. In this work, *NEP* (Fig. 7d) is measured at a bandwidth of 1 Hz. As clear from Fig. 7, R² values between simulated data and analytical results range from 0.9453 to 0.9897, confirming strong agreement across all optical characteristics. The proposed photodetector shows the peak *R*_i_ of 4.98 A/W, a peak D^*^ of 3.96 × 10^11^ cmHz^1/2^/W, and a minimum *NEP* of 2.52 × 10^–16^ W/Hz^1/2^ at cut-off wavelength. These performance metrics surpass those of recently developed InGaAs/InP^35^, HgCdTe/black phosphorus van der Waals heterojunction^41^, PbS/graphene/Al_2_O_3_/InP^60^, and HgTe colloidal quantum dots^61^-based photodetectors, which generally exhibit lower overall performance. Additionally, while the overall optical properties of the proposed photodetector are comparable to well-known bolometers^14^ commonly used in the current IR market, its superior optical response, combined with faster response times, makes it more suitable for applications requiring both high sensitivity and speed.

Furthermore, the photodetector’s photocurrent responsivity (Fig. 7b) and specific detectivity (Fig. 7c) exhibit a gradual decrease at shorter wavelengths, followed by a rapid falloff after exceeding the long wavelength cut-off point. The performance limitations of the Hg_0.7783_Cd_0.2217_Te-based photodetector are attributed to its limited absorption range, weaker built-in electric fields, and higher recombination rates. These factors result from the uniform bandgap throughout the device, which restricts wavelength sensitivity and reduces carrier separation efficiency compared to heterojunction designs. Additionally, the narrow energy bandgap of Hg_0.7783_Cd_0.2217_Te leads to higher thermal generation of carriers, increasing dark current density and noise levels. These further compromise the signal-to-noise ratio and overall photodetector performance, contributing to lower efficiency than heterojunction structures. However, despite these challenges, the detectivity of the proposed photodetector remains high compared to that of the Hg_1–*x*_Cd_*x*_Te-based photodetector in the very long wavelength IR region^55^. The photodetector exhibits maximum *R*_i_ and D^*^ at the cut-off wavelength, highlighting its capability for LWIR detection. This characteristic proves advantageous across diverse fields. The proposed photodetector technology can significantly impact industries such as thermal imaging for surveillance and safety monitoring, environmental monitoring through remote sensing of gases and pollutants, and automotive safety by improving night vision and driver-assistance systems. In defense surveillance, it enhances monitoring and target detection, while in industrial automation, it enables non-contact temperature measurements and thermal profiling for better quality control. The broad applicability of this technology across these diverse fields underscores its potential for widespread integration in scenarios demanding precise IR detection.

The observed *QE*, *R*_i_, D^*^, and *NEP* trends across varying wavelengths highlight the crucial role of optimizing the photodetector’s structure and material properties. The relationship between these parameters provides insight into how structural adjustments can enhance performance, particularly in the LWIR region. Further optimization of the device parameters, such as doping concentrations, layer dimensions, and compositional variations in the absorber layer, could improve functionality, making the photodetector even more suitable for high-performance applications. Overall, the proposed technology has the potential to significantly impact various industries where precise IR sensing is vital for optimal functionality and enhanced safety measures.

## Machine learning regression models, results, and discussion

### Evaluation of machine learning regression models

This section covers several key aspects of the machine learning approach. It introduces ML regression models, thoroughly analyzes the training and testing datasets, details the feature selection process, and presents the results. Additionally, it outlines the metrics used to assess the models’ predictive capabilities.

Machine learning techniques enable a more profound understanding of optoelectronic device behavior and underlying patterns. A fundamental ML method, regression analysis, is crucial in uncovering relationships between variables in optoelectronic devices. For example, it can reveal how changes in input factors like voltage or incident light wavelength influence electrical and optical parameters in photodetectors. These regression models quantify dependencies in the data, offering valuable insights into the critical factors governing photodetector performance.

Regression models are powerful tools that significantly reduce the time and resources needed for analyzing complex systems. These models use machine learning to accurately predict key parameters and identify missing values, making analysis more efficient. A large dataset of relevant features and target variables related to the system under study is first collected to create these models. This comprehensive data compilation is then used to train the regression model, allowing it to recognize patterns and relationships between inputs and desired outputs.

Once trained, the model can quickly predict values for new data points without running resource-intensive calculations, making it invaluable for streamlining analysis processes across various fields. This study addresses a gap in current research by providing an in-depth assessment and comparison of multiple ML regression models applied to the proposed homojunction photodetector in the LWIR spectral region. In this work, supervised ML approaches are employed, as they are well-suited for problems with clear objectives, unlike unsupervised or reinforcement learning methods^62,63^. The defined goals in supervised learning align better with research aims in fields like photodetector analysis, making it the preferred approach for such applications.

### Description and rationale of machine learning regression models

The methodology involves splitting the simulated dataset, comprising electrical and optical characteristic parameters, into separate training and testing sets. Subsequently, various regression models are employed to find the underlying trends and correlations between input variables (voltage and incident light wavelength) and output optoelectronic parameters. During the training phase, four distinct ML regression models (DT, RF, ET, and GBM) implemented in Python are applied to the simulated dataset to predict the output characteristic parameters of the photodetector. These models are specifically chosen for their robust capabilities in identifying and quantifying both linear and nonlinear relationships within complex datasets, making them particularly suitable for analyzing the diverse and complex behavior of photodetector. The rationale for selecting each model is outlined below.

#### Decision tree (DT)

DT is a widely used simple ML model for both regression and classification. DT operates by recursively dividing the dataset into branches or nodes based on specific criteria, ultimately arriving at predictions in the final leaf nodes^64^. This structure mimics human decision-making processes, making DT particularly interpretable and easy to understand. The model’s straightforward approach and ability to handle both linear and non-linear relationships have contributed to its widespread adoption in various fields, including optoelectronics. In this study, DT was selected for its capacity to provide clear insights into the relationships between input variables, such as voltage and wavelength, and the photodetector’s performance.

#### Random forest (RF)

RF is an ensemble learning model that enhances the predictive performance of individual decision trees through a bagging technique. By constructing multiple decision trees using bootstrapped samples of the dataset, RF reduces the risk of overfitting, a common issue with single decision trees. Each tree in the forest is trained on a random subset of predictors, thereby reducing the correlation between trees and increasing randomness. This leads to improved generalization and robustness in prediction^64,65^. In this study, RF was chosen for its proven ability to handle high-dimensional, non-linear datasets, and its robustness in predicting the optoelectronic parameters of the photodetector.

#### Extra trees (ET)

ET is another ensemble learning model derived from the RF model. Like RF, ET also builds multiple decision trees, but with a key difference: ET uses the entire training dataset and selects splitting points at random for each node, which introduces greater randomness into the model. This randomization helps reduce both bias and variance, making ET more resistant to overfitting^64^. Additionally, ET grows fully-developed trees that account for all possible splitting points, which enhances the precision of the model. ET was chosen for its ability to handle complex, non-linear relationships with improved accuracy and generalization compared to other ensemble methods.

#### Gradient boosting machine (GBM)

GBM is a versatile and powerful ensemble learning model that combines multiple weak learners, typically decision trees, to form a strong predictive model capable of excelling in both regression and classification tasks. GBM constructs predictive models using an iterative process of back-fitting and non-parametric regression techniques. Instead of creating a single comprehensive model at once, GBM starts with a simple initial model and progressively builds upon it, with each new model in the ensemble correcting the errors of the previous one. This sequential process allows GBM to gradually improve overall predictive performance, effectively handling complex and non-linear data relationships^66^. In this study, GBM was selected for its ability to capture the subtle and intricate interactions between input variables, such as applied voltage and light wavelength, and key performance metrics like photocurrent and dark current. The iterative nature of GBM enables it to refine its predictions over time, achieving high accuracy. This makes GBM an excellent choice for optimizing the performance of optoelectronic devices, where precision is critical to evaluating device behavior and performance.

### Hyperparameters and their significance

Hyperparameters are key in ML regression algorithms, affecting their predictive performance, generalization ability, and overfitting prevention. For the DT, RF, ET, and GBM regressors, all key hyperparameters such as maximum depth (max_depth), minimum sample split (min_samples_split), minimum samples leaf (min_samples_leaf), and number of estimators (n_estimators) are set to their default values as shown in Table 2, aiming to optimize the prediction performance of the proposed photodetector. Table 2 also lists the significance of each hyperparameter. Adjusting these hyperparameters can significantly impact model performance and training time for all the ML regression models. Increasing the complexity of the models (through deeper trees or more estimators in ensemble models) generally leads to better performance up to a point, but also increases computational cost. However, beyond a certain threshold, further increases in complexity may lead to minimal additional improvements in performance and potentially increase the risk of overfitting.

**Table 2:** Default hyperparameters and their significance for proposed ML regression models.

Hyperparameter	Values				Significance of the parameter
	DT	RF	ET	GBM	
max_depth	None	None	None	None	The max_depth parameter in tree-based models is crucial as it controls the model’s complexity, balancing its ability to capture complex patterns in the data against the risk of overfitting, thereby directly influencing the model’s capacity to generalize to new, unseen data The max_depth parameter is set to None by default to allow trees to grow without depth restrictions, maximizing flexibility but potentially increasing overfitting risk, thus leaving depth control to the user’s judgment based on their specific needs and dataset characteristics
min_samples_split	2	2	2	2	The parameters min_samples_split and min_samples_leaf determine the smallest number of samples necessary to divide an internal node or constitute a leaf node, respectively They help to prevent the model from learning very specific rules for individual samples These default values (min_samples_split = 2, min_samples_leaf = 1) allow for maximum tree growth and granularity, enabling the model to capture specific patterns in the data. However, this flexibility can lead to overfitting, especially in smaller datasets, so these parameters are often adjusted to control model complexity and improve generalization
min_samples_leaf	1	1	1	1	
n_estimators	–	100	100	100	This determines the number of trees in the ensemble, affecting the model’s robustness and performance. The n_estimators parameter usually controls the number of individual models combined in an ensemble method. This setting is common in techniques that utilize multiple, often tree-based, predictors to enhance overall predictive power The default value of 100 for n_estimators in RF, ET, and GBM is a compromise between model performance and computational efficiency. It typically provides a good balance of predictive power and training time for many datasets, offering substantial improvements over single trees without excessive computational burden. However, the optimal number can vary depending on the problem and dataset

**Fig. 8:**
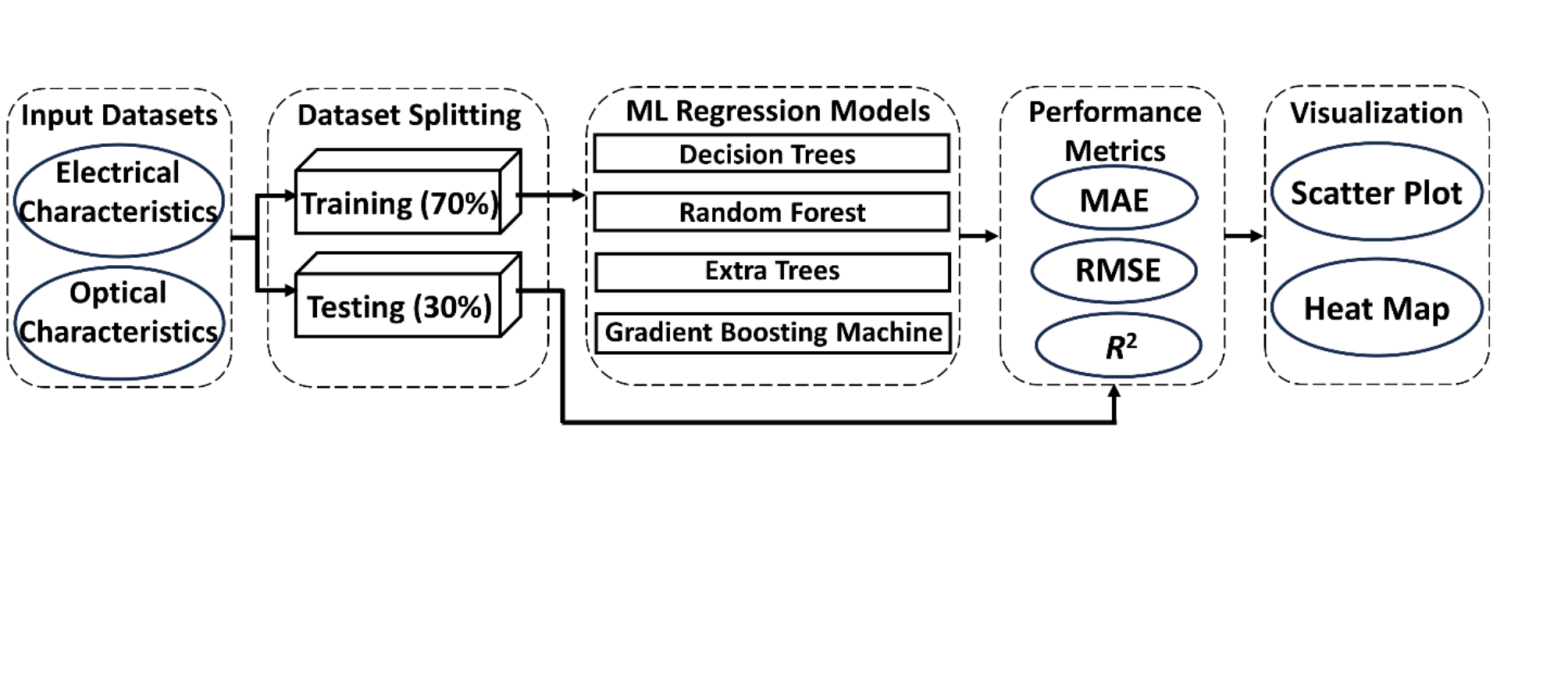
Workflow of the ML-driven predictive process of the p^+^-Hg_0.7783_Cd_0.2217_Te/n^–^-Hg_0.7783_Cd_0.2217_Te LWIR homojunction photodetector.

### Model validation and metrics

To rigorously validate the simulation results and assess the performance of the ML regression models, three key statistical metrics were employed: mean absolute error (MAE), root mean squared error (RMSE), and *R*-squared (*R*²) value. These metrics serve distinct yet complementary roles in the evaluation process. The MAE calculates the average magnitude of differences between the model’s predictions and the actual observed values, offering an easily interpretable measure of the model’s accuracy. The RMSE quantifies the standard deviation of the model’s prediction errors, with higher values indicating less accurate predictions. *R*² value quantifies how well the model explains the variability in the target variable, with values closer to 1 indicating a better fit. The following equations are employed to calculate these performance metrics^64^

42 $$\:\text{M}\text{A}\text{E}=\frac{1}{N}\sum\:_{i=1}^{N}\left(\frac{\text{Actual}{\text{\_Value}}_{i}-{\text{Predicted\_Value}}_{i}}{\text{Actual}{\text{\_Value}}_{i}}\right)$$

43 $$\:\text{R}\text{M}\text{S}\text{E}=\sqrt{\frac{1}{N}\sum\:_{i=1}^{N}{\left(\text{Actual}{\text{\_Value}}_{i}-{\text{Predicted\_Value}}_{i}\right)}^{2}}$$

44 $$\:{R}^{2}=1-\frac{\sum\:_{i=1}^{N}{\left({\text{Predicted\_Value}}_{i}-{\text{Actual\_Value}}_{i}\right)}^{2}}{\sum\:_{i=1}^{N}{\left({\text{Actual\_Value}}_{i}-{\text{Average\_Target\_Value}}_{i}\right)}^{2}}$$

where *N* is the total number of data samples employed for validating the performance of the ML regression model.

By applying these metrics across various test set sizes (30–70% of the total dataset), it was possible to comprehensively assess each model’s predictive accuracy and robustness under different data allocation scenarios. This approach not only allowed for comparison of the performance of different ML models (DT, RF, ET, and GBM) but also validated the simulation results by demonstrating the models’ ability to accurately capture the underlying physics of the photodetector. The consistently low MAE and RMSE values, coupled with high *R*² values (often reaching 1.0), across different test sizes, provide strong evidence for the validity and reliability of the simulation approach. To assess model robustness, multiple data splitting strategies were utilized for ML regression models. These models were trained on varying proportions of the dataset: 70%, 60%, 50%, 40%, and 30%. Correspondingly, the remaining portions (30%, 40%, 50%, 60%, and 70%) were used for testing. This approach allowed for a comprehensive evaluation of the ML regression models’ performance across different ratios of training to testing data, providing insights into model behavior under various data allocation scenarios. The complete workflow of the ML-driven predictive process for the proposed photodetector is illustrated in Fig. 8.

### Results and discussion

**Fig. 9:**
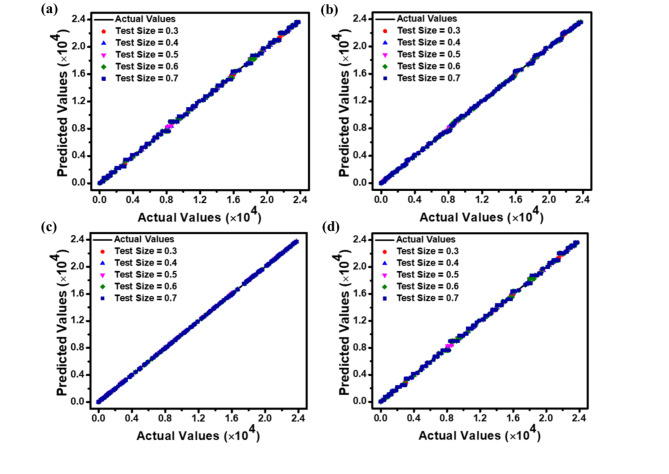
Scatter plot visualizations showing correlations between predicted and simulated *J*_dark_ values for (**a**) DT, (**b**) RF, (**c**) ET, and (**d**) GBM regression models, across various test set sizes.

**Fig. 10:**
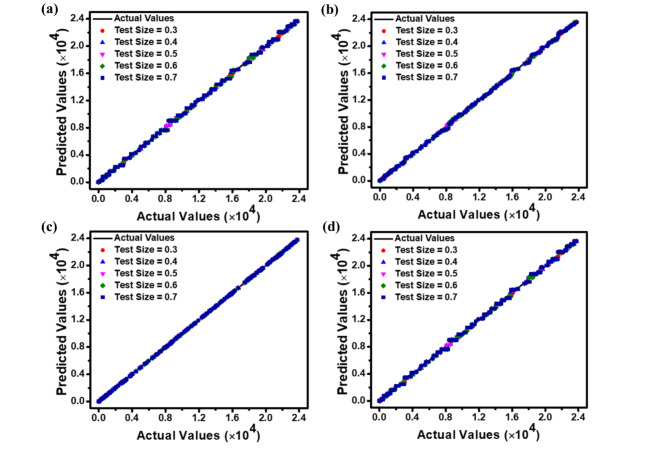
Scatter plot visualizations showing correlations between predicted and simulated *J*_light_ values for (**a**) DT, (**b**) RF, (**c**) ET, and (**d**) GBM regression models, across various test set sizes.

**Fig. 11:**
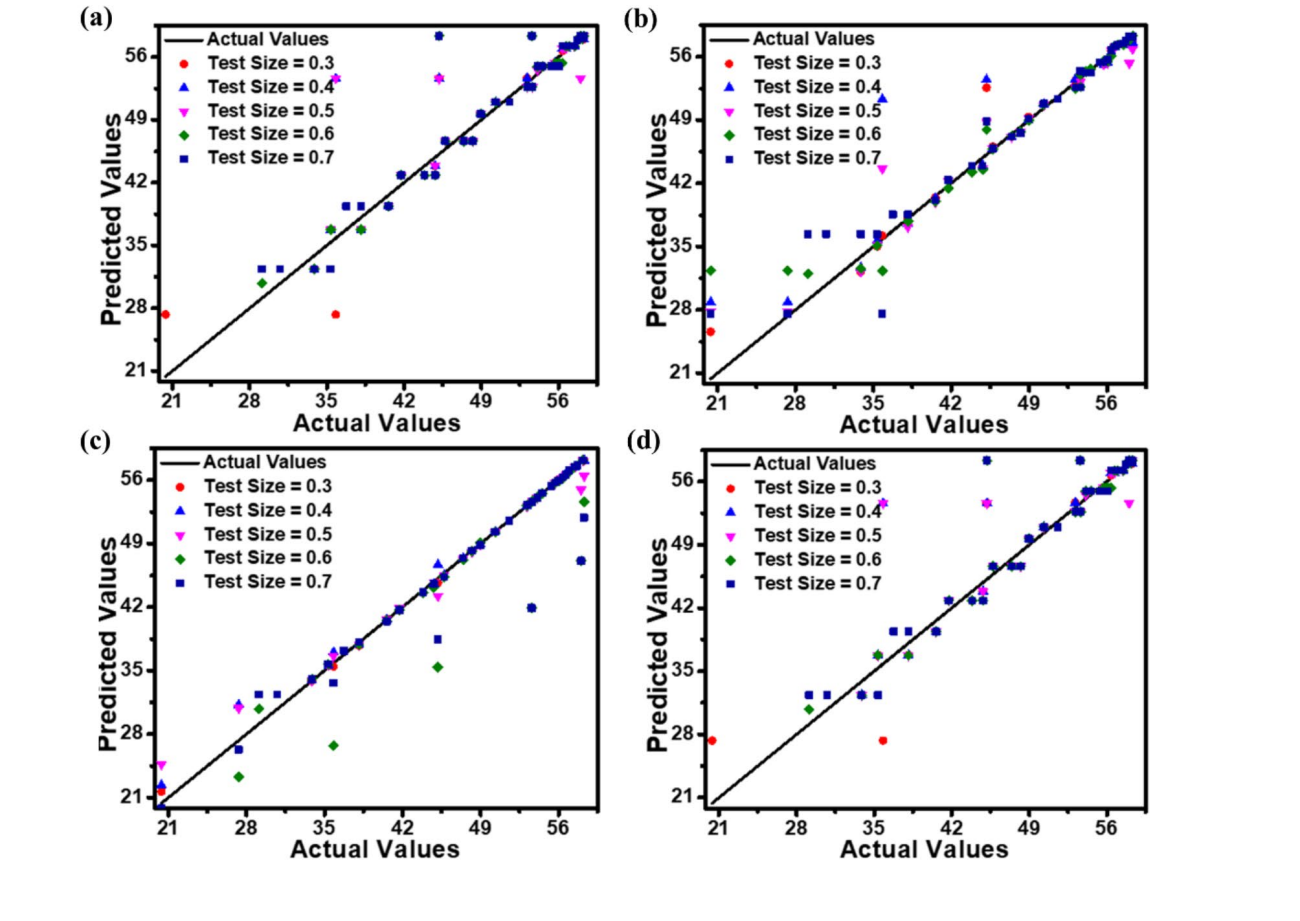
Scatter plot visualizations showing correlations between predicted and simulated *QE* values for (**a**) DT, (**b**) RF, (**c**) ET, and (**d**) GBM regression models, across various test set sizes.

**Fig. 12:**
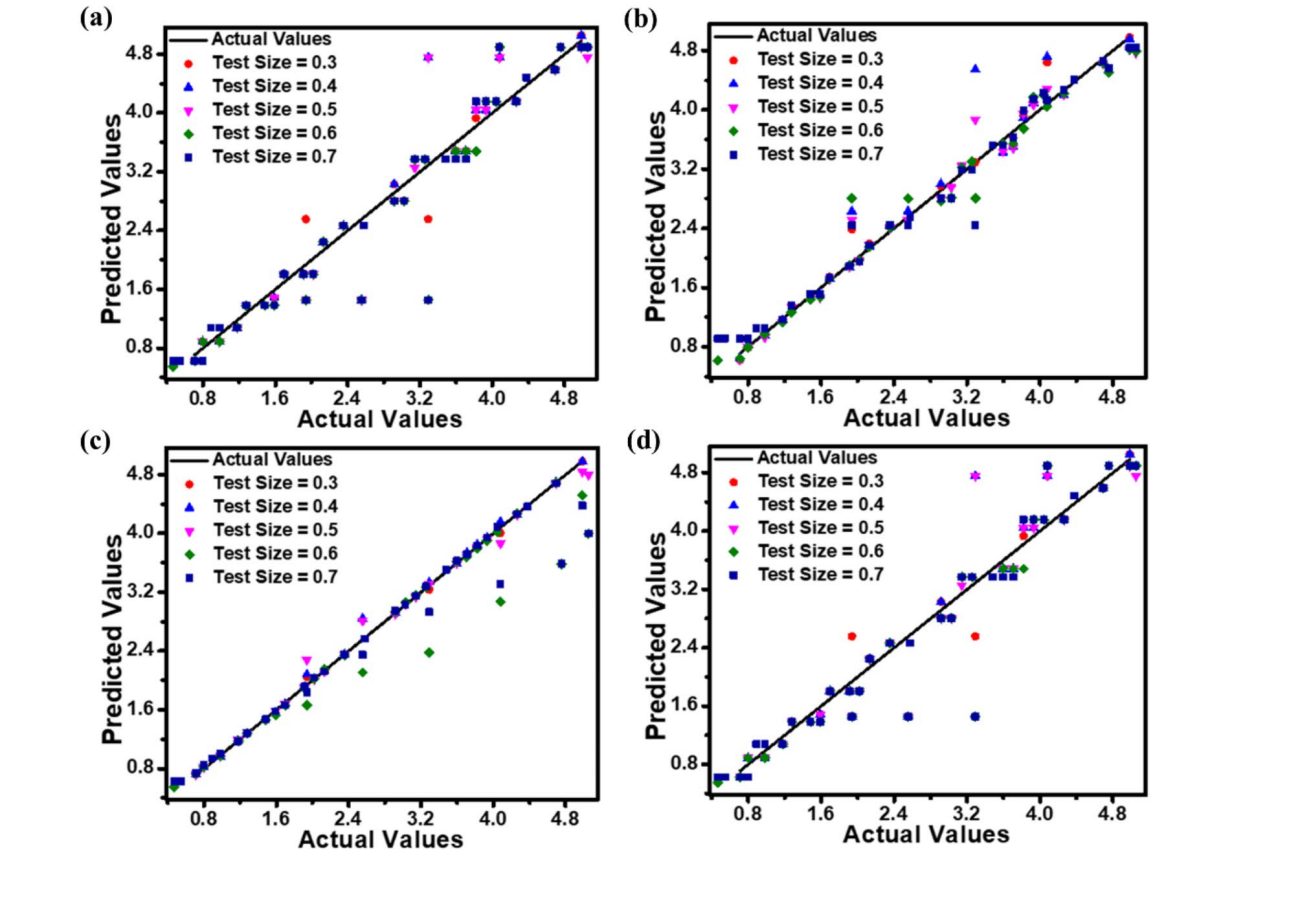
Scatter plot visualizations showing correlations between predicted and simulated *R*_i_ values for (**a**) DT, (**b**) RF, (**c**) ET, and (**d**) GBM regression models, across various test set sizes.

The correlations between predicted and actual values for *J*_dark_, *J*_light_, *QE*, and *R*_i_ are illustrated as scatter plots in Figs. 9, 10 and 11, and 12, respectively. These plots compare the predictions of DT, RF, ET, and GBM ML regression models against experimental data. The plots illustrate how the model performs when evaluated on different-sized test sets, which comprise between 30% and 70% of the entire dataset. Incorporating this range of test sizes allows for a comprehensive analysis of the model’s effectiveness across different training ratios to testing data. These scatter plots visually represent each model’s accuracy, performance, and general trends across different test sample sizes. Figures 9, 10, 11 and 12 suggest that as the test dataset expands, there is an increasing divergence between the model’s predictions and the actual results. This phenomenon may be attributed to the model’s tendency to overfit the training data and its difficulty in accommodating the increased diversity found in more extensive test datasets. This observation provides insight into the models’ behavior and limitations as the proportion of testing data increases.

Among the four models examined, the ET regression model tends to outperform DT, RF, and GBM in accurately predicting the photodetector’s optoelectronic parameters. This enhanced performance can be ascribed to the ET model’s implementation of highly randomized splitting criteria and increased overall randomization. This approach results in a more diverse set of decision trees within the ensemble, enabling the model to effectively capture diverse data patterns while mitigating the risk of overfitting.

**Fig. 13:**
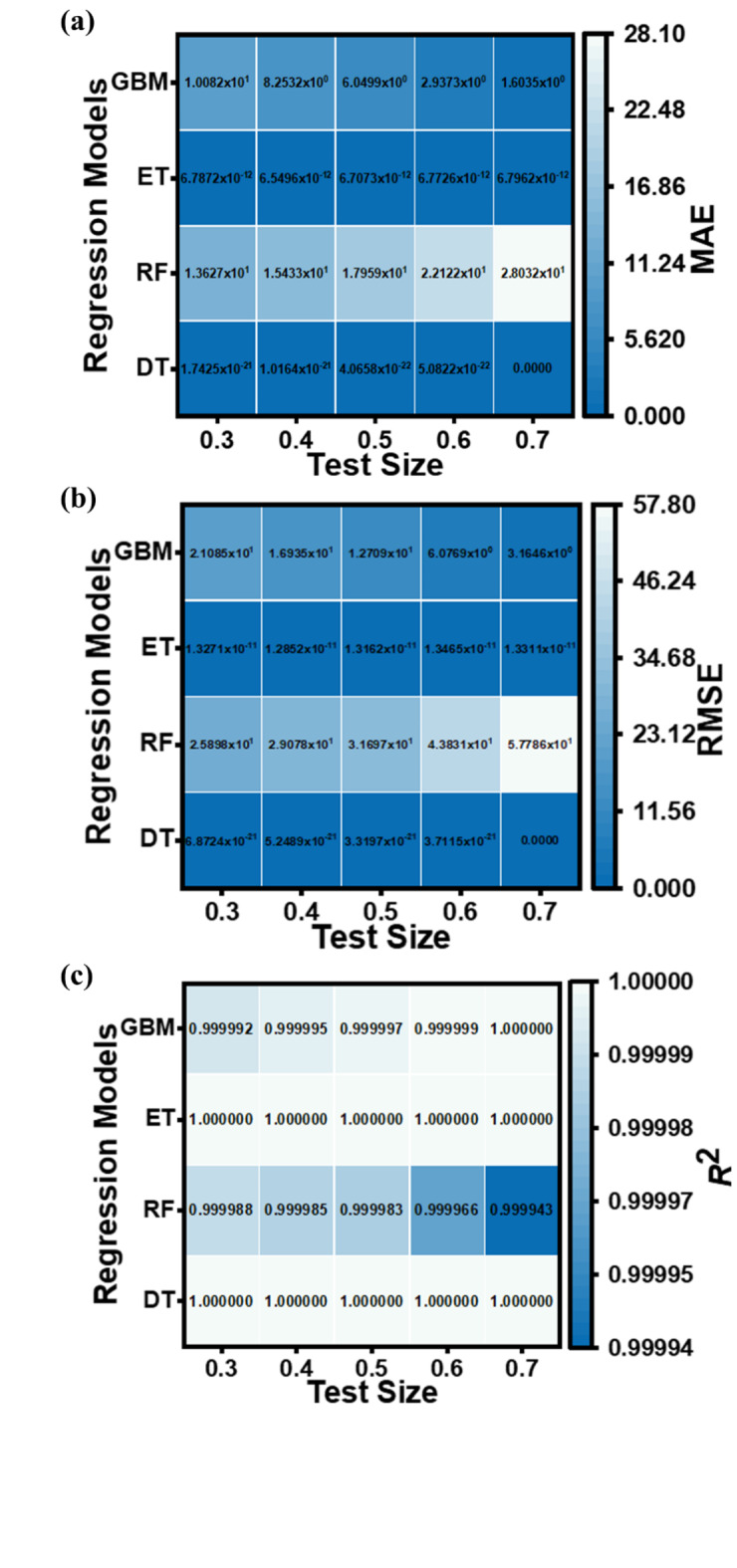
Heatmap of performance evaluation of DT, RF, ET, and GBM ML regression models for predicting *J*_dark_ values with test sizes varying from 30–70% based on (**a**) MAE, (**b**) RMSE, and (**c**) *R*^2^ values.

**Fig. 14:**
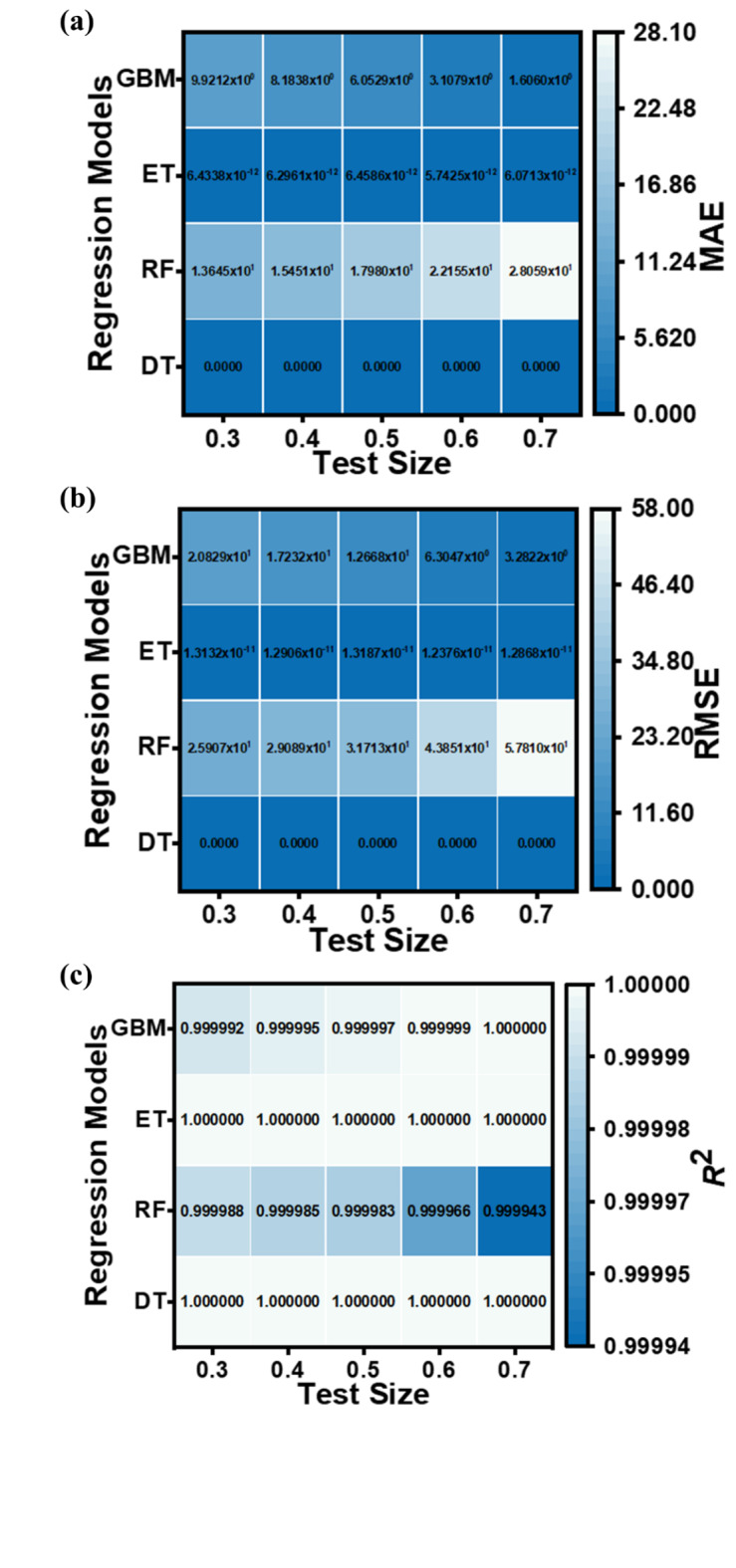
Heatmap of performance evaluation of DT, RF, ET, and GBM ML regression models for predicting *J*_light_ values with test sizes varying from 30–70% based on (**a**) MAE, (**b**) RMSE, and (**c**) *R*^2^ values.

**Fig. 15:**
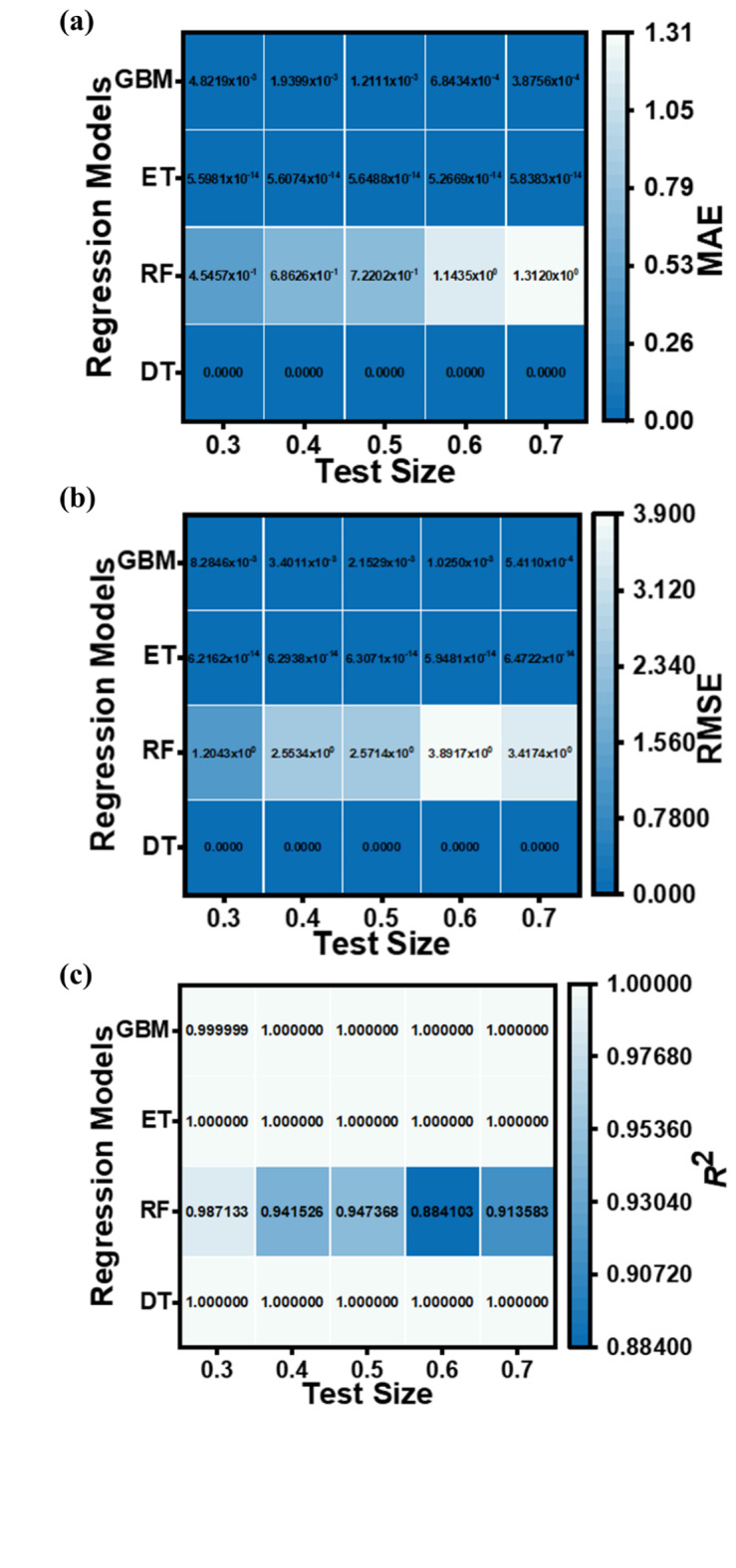
Heatmap of performance evaluation of DT, RF, ET, and GBM ML regression models for predicting *QE* values with test sizes varying from 30–70% based on (**a**) MAE, (**b**) RMSE, and (**c**) *R*^2^ values.

**Fig. 16:**
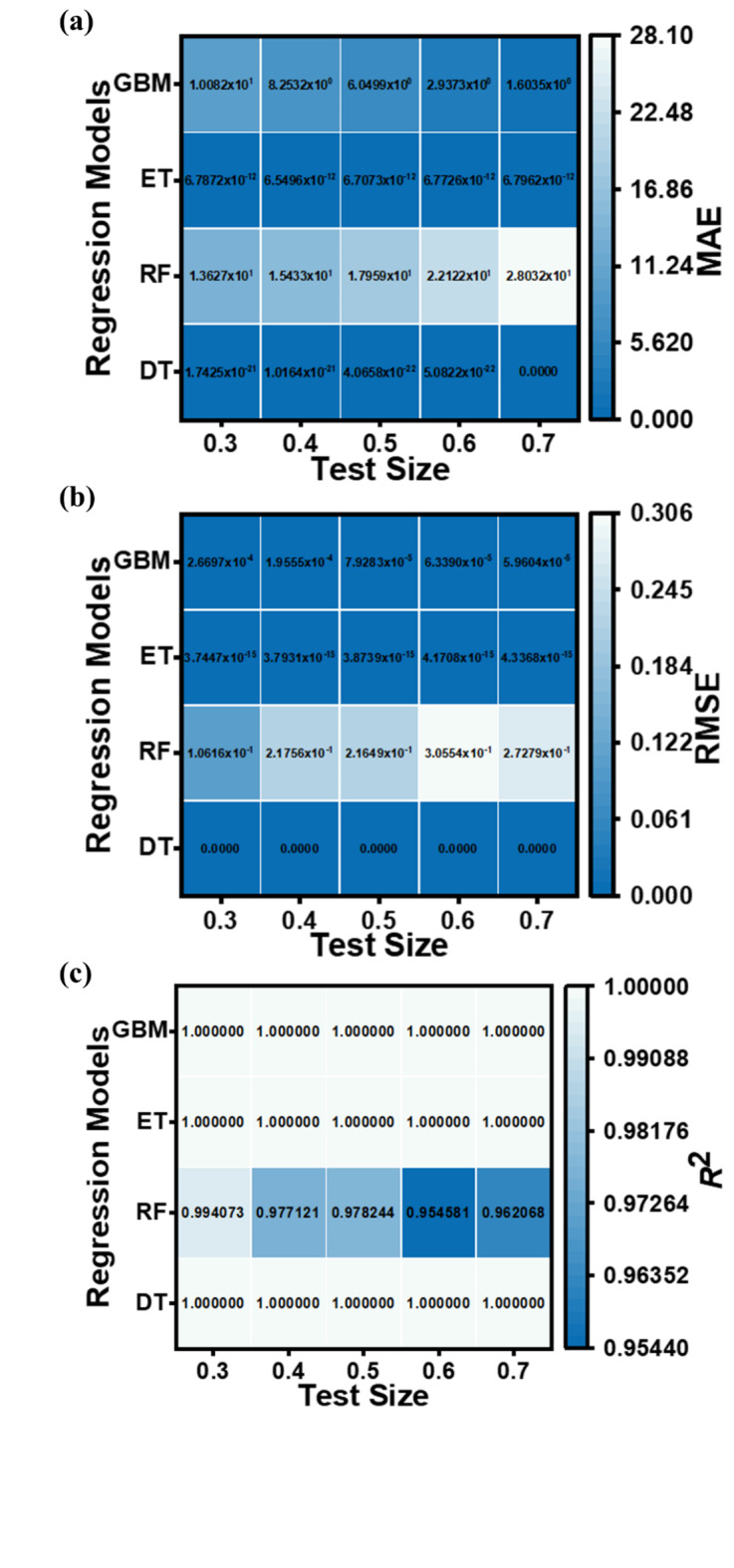
Heatmap of performance evaluation of DT, RF, ET, and GBM ML regression models for predicting *R*_i_ values with test sizes varying from 30–70% based on (**a**) MAE, (**b**) RMSE, and (**c**) *R*^2^ values.

The performance metrics (MAE, RMSE, and *R*^2^ values) for the applied ML regression models are shown in heatmap Figs. 13, 14, 15 and 16. These metrics evaluate the models across various test set sizes to predict the optoelectronic characteristics of the proposed LWIR photodetector and assess the discrepancies between predicted and actual parameter values to indicate model accuracy.

Analysis of Figs. 13, 14, 15 and 16 indicates that the DT and ET regression models consistently demonstrate low MAE and RMSE values. These models achieve a perfect *R*² value of 1 across all evaluated test set sizes. This optimal *R*² value suggests that the DT and ET models explain nearly all data variance, indicating an exceptional correlation between predicted and observed photodetector parameters. The RF and GBM regression models also demonstrated strong performance, yielding comparably low error metrics in their predictions.

The models’ robust predictive capabilities are evidenced by their consistently low error metrics (MAE and RMSE) and high *R*² values, even when trained on limited data subsets. These findings highlight the efficacy of DT and ET regression models in this context. A detailed examination of the results reveals that a test size of 30% yields optimal performance across the evaluated test sizes, suggesting that a more compact test set offers a more accurate evaluation of model predictive capability. Furthermore, they emphasize the importance of selecting appropriate modeling techniques based on specific dataset characteristics and required prediction accuracy. These findings highlight the diverse strengths of various ML regression models and underscore the critical need to choose a model that aligns with both the dataset’s unique features and the desired precision in predictions.

## Overall discussion

The results of this study demonstrate the significant potential of Hg_1–*x*_Cd_*x*=0.2217_Te-based LWIR homojunction photodetector, particularly for high-sensitivity, low-noise, and high-speed applications. The demonstrated *J*_dark_ of 0.20 mA/cm² and *QE* of 58.30% at 77 K indicate that the device is well-suited for use in high-frequency systems. The high cut-off frequency of 104 GHz and the fast response time of 0.8 ps make this photodetector highly competitive in optical communication and IR imaging systems, where speed and sensitivity are critical. The photodetector’s low noise characteristics further enhance its performance in these high-precision environments, making it a promising candidate for defense surveillance, environmental monitoring, and high-speed optical switching.

The integration of ML techniques into the study provides a robust framework for predicting the optoelectronic performance of the device. By employing ML regression models, the research offers a more efficient means of optimizing device design and predicting performance characteristics. These models demonstrate strong predictive capabilities, with high *R*² values and low error metrics across various test set sizes. The ability to predict key parameters such as *J*_dark_, *J*_light_, *QE*, and *R*_i_ without resorting to resource-intensive simulations marks a significant advancement in photodetector research, enabling faster and more accurate design optimization.

However, despite these promising findings, several limitations must be addressed to improve the practical applicability of the proposed photodetector. One of the most significant limitations is the requirement for cryogenic cooling to maintain low dark current levels. At room temperature, *J*_dark_ increases substantially due to thermal excitation, reaching levels that can equal or exceed the *J*_light_. This restricts the photodetector’s use in commercial and portable applications, where cryogenic cooling is often impractical due to increased cost, complexity, and power consumption.

To overcome the limitation of high dark current at room temperature, heterojunction designs or barrier architectures emerge as promising solutions. These alternative architectures could enable higher-temperature operation without significantly sacrificing performance, offering a promising avenue for improving device usability. The integration of advanced materials, such as 2D materials like graphene and black phosphorus could further enhance the performance by improving carrier mobility and reducing recombination losses. Exploring these architectures could expand the practical applications of Hg_1–*x*_Cd_*x*_Te-based photodetectors, particularly in portable and low-power systems where cooling mechanisms are impractical.

Another limitation is the dependence on numerical simulations and ML regression models without experimental validation. Real-world fabrication challenges, such as material defects and interface traps, could cause discrepancies between simulated and actual performance. Future work should prioritize experimental validation to bridge this gap and refine device designs.

## Conclusions

The research demonstrates the potential of Hg_1–*x*_Cd_*x*=0.2217_Te for developing LWIR homojunction photodetectors, evaluated comprehensively through theoretical analysis, simulation, and machine. The performance of the photodetector was assessed at a wavelength of 10.6 μm and a temperature of 77 K. Results from Silvaco TCAD simulation were compared with analytically derived data, showing good agreement and confirming the validity of the considered model. The photodetector exhibited a low dark current density of 0.20 mA/cm^2^, a high photocurrent density of 4.98 A/cm^2^, a quantum efficiency of 58.30%, a peak photocurrent responsivity of 4.98 A/W, a specific detectivity of 3.96 × 10^11^ cmHz^1/2^/W, and a noise equivalent power of 2.52 × 10^–16^ W/Hz^1/2^. The 3-dB cut-off frequency of 104 GHz and a rise time of 0.8 ps further underline its suitability for high-speed optoelectronic systems.

Additionally, a comparative study of four machine learning regression models (DT, RF, ET, and GBM) was conducted to streamline the design process. The DT and ET models demonstrated strong predictive accuracy, with *R*^2^ values reaching up to 1 and low error metrics across various test sets. These models provide reliable predictions for essential optoelectronic parameters, offering a robust framework for future design optimization and performance improvements. These findings emphasize the potential of HgCdTe-based homojunction photodetectors for applications requiring low-noise, high-speed, and high-sensitivity detection, particularly in the LWIR range. By integrating simulation and machine learning, this study provides valuable insights into performance optimization for next-generation infrared photodetectors, with potential applications in infrared sensing and high-speed optical communication.

## Data Availability

All data generated or analyzed during this study are included in this article.
